# Loss of Rab8a‐Dependent Tethering of Lipid Droplets to Mitochondria Contributes to Hypoxia–Reoxygenation Injury

**DOI:** 10.1002/mco2.70845

**Published:** 2026-08-06

**Authors:** Jialing Tang, Nathan Joyce, Thomas Laval, Eddie Tam, Hye Kyoung Sung, Mireille Ouimet, Gary Sweeney

**Affiliations:** ^1^ Department of Biology, Faculty of Science York University Toronto Ontario Canada; ^2^ Department of Biochemistry, Microbiology and Immunology, Faculty of Medicine University of Ottawa Heart Institute Ottawa Ontario Canada

**Keywords:** adiponectin, cardiomyocyte, hypoxia, ischemia, lipophagy, lipolysis, lipid droplet, mitochondria, reoxygenation

## Abstract

Tethering of lipid droplets (LDs) to mitochondria facilitates fatty acid oxidation and is a key adaptive response that helps cardiomyocytes cope with hypoxia–reoxygenation (HR) stress. However, the molecular mechanisms regulating these interactions during ischemia are not fully understood. We found that HR significantly decreased cardiomyocyte expression and phosphorylation of Rab8a, a small GTPase that interacts with the LD‐associated protein perilipin 5 to mediate LD–mitochondria tethering. Using long‐chain fatty acid (LCFA) tracking assays we confirmed that loss of Rab8a disrupted physical contact between these organelles to impair the transfer of LCFA from LDs to mitochondria. Furthermore, HR led to the accumulation of intracellular lipids via a combination of both lipolysis and lipophagy, resulting in metabolic stress. To counteract this defect, we evaluated the therapeutic potential of ALY688, a peptide agonist of adiponectin receptors known to stimulate the AMPK–Rab8a signaling axis. Our data indicated that treatment with ALY688 preserved Rab8a expression during HR, thereby maintaining LD–mitochondria interactions and improving metabolic function. These findings uncover a novel Rab8a‐dependent mechanism regulating lipid metabolism under ischemic/hypoxic stress and highlight the potential of adiponectin mimetics like ALY688 to mitigate metabolic dysfunction in ischemic heart disease.

## Graphical abstract

Rab8a‐dependent tethering of lipid droplets to mitochondria sustains fatty acid oxidation and cardiomyocyte metabolic flexibility under normoxia. During hypoxia/reoxygenation, Rab8a loss and PLIN5‐associated lipophagy uncouple lipid breakdown from mitochondrial utilization, driving lipotoxicity and cell death. The adiponectin receptor agonist ALY688 preserves AMPK–Rab8a–PLIN5‐mediated lipid droplet–mitochondria contact, maintaining fatty acid oxidation, to mitigate hypoxia/reoxygenation‐induced cardiomyocyte injury. *The graphic abstract was created with BioRender.com*

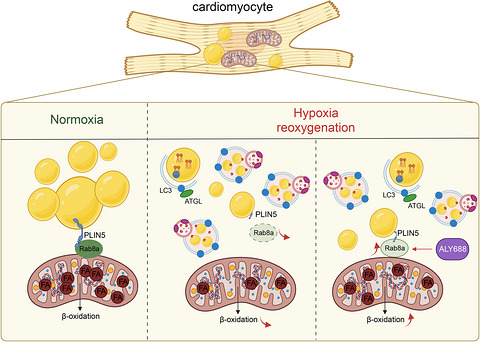

## 1 Introduction

The heart has a substantial energy demand, requiring the production of large amounts of ATP to support its contractile function [1]. ATP is primarily generated through mitochondrial oxidation of various substrates, including fatty acids, glucose, lactate, ketones, and amino acids [2]. A healthy heart is metabolically flexible, capable of switching between fatty acids and other energy sources like glucose, depending on factors such as workload, neurohormonal signals, and fuel availability. In contrast, heart failure is associated with significant alterations in cardiac energy metabolism, resulting in a metabolically compromised heart, reduced cardiac efficiency, and progressive contractile dysfunction [3, 4, 5]. Changes in cardiac metabolism during ischemia/reperfusion (I/R) injury are well documented, which include fatty acid oxidation [6] and uncoupling of glycolysis from glucose oxidation [7]. Adult mammalian cardiomyocytes have high energy demands. Under fasting conditions, they obtain about 70% of their energy from the oxidation of long‐chain fatty acids (LCFA) [8].

Lipid droplets (LDs) are intracellular organelles that mainly store triacylglycerol (TAG) and sterol esters as energy reserves. During starvation, cells shift from glycolysis to fatty acid oxidation. This occurs by breaking down LDs and transporting free fatty acids (FFAs) to mitochondria for β‐oxidation. The breakdown happens via enzymatic hydrolysis by neutral lipases (classical lipolysis) or through a selective form of autophagy called lipophagy [9]. Both processes are tightly regulated by members of the perilipin (PLIN) family of LDs proteins [10]. In addition to serving as a nutrient source, excessive cytoplasmic FFAs can be harmful by generating reactive oxygen species (ROS), which can damage mitochondria and other cellular components [11]. Lipophagy may provide a cytoprotective role by breaking down lipids to sustain energy supply, which helps prevent necrosis, apoptosis, and ATP depletion facilitated by mitochondrial dysfunction. It also promotes the metabolism of potentially lipotoxic molecules [11]. Disrupted lipid metabolism, especially imbalanced lipolysis and lipophagy, is a key factor in the development of many heart diseases. The heart mainly uses fatty acids to produce energy. When the balance between lipid uptake and oxidation is disrupted, lipids accumulate excessively. This buildup contributes to cardiac lipotoxicity and eventually leads to myocardial dysfunction [12]. In healthy hearts, lipolysis and lipophagy function synergistically to regulate LD catabolism, where lipolysis breaks down large LDs into smaller ones that can be effectively degraded through lipophagy [8, 13]. However, conditions such as obesity, diabetes, and heart failure disrupt these processes. This disruption creates a harmful feedback loop. Lipolysis becomes dysregulated, and lipophagy is impaired. As a result, LDs accumulate excessively, and inflammation persists [12, 14]. This metabolic imbalance leads to the buildup of toxic lipid intermediates, especially ceramides (Cers) and diacylglycerol (DAG). Unlike neutral TAGs, these molecules damage cells. They contribute to cardiac steatosis, impair mitochondrial function, and drive the progression of heart failure [15].

Cardiomyocyte LD turnover is tightly controlled by membrane contact sites (MCSs). These structures coordinate lipid trafficking between LDs, mitochondria, lysosomes, and the endoplasmic reticulum (ER). This coordination maintains metabolic balance and prevents lipotoxicity. Mitochondria–LD tethering is mediated by proteins such as PLIN5 and the Mfn2/Hsc70 complex. This tethering enables direct fatty acid transfer from LDs to mitochondria for β‐oxidation. The process is essential for cardiac energy production. PLIN5 deficiency has been shown to impair this mechanism [16, 17, 18, 19]. Rab8a (Ras‐related protein 8A), a small GTPase, further stabilizes ER–LD–mitochondria tripartite junctions, supporting phospholipid exchange, LDs expansion, and mitochondrial membrane integrity [20, 21, 22, 23]. In parallel, lysosome–LD contacts, regulated by Annexin A4 and Rab7, mediate lipophagic degradation, while Rab8a also modulates lysosomal positioning to facilitate lipid hydrolysis [24, 25]. In heart failure, disruption of these contact sites broadly impairs cardiac lipid metabolism. LD–mitochondria proximity is reduced, and fatty acid oxidation declines. While Rab8a deficiency worsens phospholipid imbalance and lipotoxicity by disrupting ER–LD–mitochondria junctions and altering lysosomal positioning [26, 27]. This underscores the central role of organelle tethering networks in maintaining cardiac lipid homeostasis and driving the development of metabolic cardiomyopathies.

Adiponectin, an adipokine secreted predominantly by adipose tissue, exerts pleiotropic cardioprotective effects in cardiometabolic diseases. These effects occur through activation of its receptors, AdipoR1 and AdipoR2, and downstream signaling pathways, mainly the AMPK pathway [28]. During I/R injury, adiponectin attenuates myocardial damage by suppressing oxidative stress through upregulation of antioxidant enzymes, inhibiting inflammatory cytokine production, and reducing cardiomyocyte apoptosis [29, 30]. Synthetic adiponectin receptor agonists, such as the small molecule AdipoRon and the peptide mimetic ALY688, mimic these protective effects in preclinical models. Studies show they improve left ventricular function, reduce infarct size, and lessen pathological remodeling after myocardial infarction [31, 32]. At the subcellular level, adiponectin regulates LDs dynamics through AMPK activation, facilitating lipid mobilization during metabolic stress [33]. In cardiomyocytes, adiponectin improves mitochondrial function. It promotes assembly of electron transport chain complexes, optimizes membrane potential, and increases ATP production. These actions protect the heart from lipotoxicity and energy loss during hypoxic stress [34]. Recent evidence reveals a mechanistic link between adiponectin signaling and LD–mitochondria coupling via the AMPK–Rab8a axis. Adiponectin activates AMPK, which increases GTP‐bound active Rab8a. Active Rab8a binds to PLIN5, promoting LD–mitochondria interactions which facilitating fatty acid transfer for mitochondrial oxidation [20]. This mechanism is supported by studies showing that metformin, which activates AMPK similarly to adiponectin, modulates Rab8a expression in regulating LDs dynamics, and inhibition of AMPK signaling attenuates Rab8a expression [35]. Together, these findings suggest that adiponectin serves as a critical regulator of cardiometabolic homeostasis by coordinating LD–mitochondria interactions through AMPK‐dependent mechanisms.

Although the cardiac protective functions of adiponectin are widely characterized, little is known about adiponectin's roles in I/R, and the subcellular mechanism whereby it protects against I/R. Here, we used ALY688 as an adiponectin mimetic to elucidate these mechanisms. We demonstrate that during hypoxia/reoxygenation (H/R) in cardiomyocytes, LDs content decreased, primarily via lipophagy‐mediated lipolysis, producing excessive amounts of FFAs. Rab8a activity was decreased by H/R, resulting in reduced β‐oxidation of produced FFAs and lipotoxicity. Our results reveal that upregulation of Rab8a expression by the adiponectin receptor agonist ALY688 during H/R protects against lipotoxicity contributed by the uncoupling of LDs breakdown from fatty acid utilization.

## 2 Results

### 2.1 Hypoxia–Reoxygenation Altered the Lipidome of H9c2 Cells and Decreased LD Content

**FIGURE 1:**
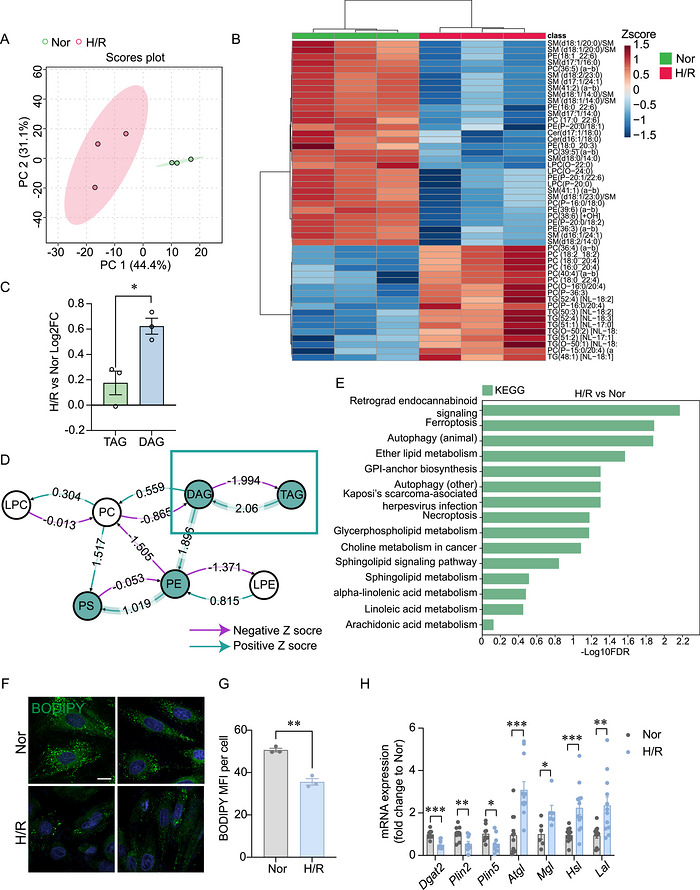
H/R reduced LD content in H9c2 cells. (A) PCA of lipidomics analysis in H9c2 cells subjected to normoxia or H/R. (B) Heat map hierarchical clustering of lipidomics analysis of H9c2 cells subjected to normoxia or H/R. (C) TAG and DAG level in H9c2 cells subjected to normoxia or H/R. (D) Lipidomic pathway analysis by BioPan. *Abbreviations*: LPC: lysophosphatidylcholine; PC: phosphatidylcholine; PS: phosphatidylserine; PE: phosphatidylethanolamine; LPE: lysophosphatidylethanolamine. (E) KEGG pathway enrichment analysis of significant lipids revealed by lipidomics analysis. (F and G) Representative images of BODIPY staining and quantification comparing normoxia and H/R in H9c2 cells. (H) mRNA expression of DGAT2, PLIN2, PLIN5, ATGL, MGL, HSL, LAL in H9c2 cells subjected to normoxia or H/R. Scale bar: 10 µm. Results are presented as mean ± SEM (*n* = 3–9 per group). *p < 0.05, **p < 0.01, ***p < 0.001 versus normoxia control. One‐way ANOVA was run for statistical analysis.

Hypoxia incubation was first validated by the activation of HIF1⍺ (Figure S1A,B). To better understand lipid composition changes induced by H/R in rat cardiomyocyte H9c2 cells, we performed a comprehensive lipidomic analysis. Principal component analysis (PCA) and hierarchical clustering heatmaps revealed distinct lipid species profiles between normoxia and H/R conditions in H9c2 cells (Figure 1A,B). Further analysis identified DAG as one of the most significantly increased glycolipid classes in H/R cells (Figure 1C). Additionally, Cer (d18:1/16:0) was significantly elevated under H/R conditions compared with normoxia and is often considered a primary mediator of insulin resistance and lipotoxicity. Cer (d18:1/18:0) and Cer (d18:1/24:1), which have been positively associated with cardiovascular mortality in clinical studies, were also significantly upregulated in response to H/R stress (Figure S1C–E). Lipidomic pathway analysis using BioPan [36] demonstrated marked activation of enzymatic reactions involved in the hydrolysis of TAG into DAG (Figure 1D). To further explore lipid pathways enriched under H/R conditions, we utilized the KEGG database, which identified autophagy, a crucial process for cellular adaptation to metabolic stress and clearance of damaged components, as being upregulated under H/R (Figure 1E). Given the role of LDs as primary storage sites for TAG, we hypothesized that LD dynamics might be altered under H/R. To test this, we measured LD content in both normoxia and H/R conditions. H/R significantly reduced LD content, as indicated by decreased mean fluorescence intensity (MFI; Figure 1F,G), reduced LD number (Figure A1F), though no change in LD size (Figure S1G). Since DAG can be converted to TAG by DAG acyltransferases (DGATs) and stored in LDs, and LDs are coated by PLIN family proteins (PLIN1–PLIN5) that stabilize LDs and protect them from lipase activity, we next examined gene expression changes. During H/R, we observed significant decreases in *Dgat2*, *Plin2*, and *Plin5* mRNA levels (Figure 1H), while DGAT1, PLIN1, PLIN3, and PLIN4 remained unchanged (Figure S1H–K). Finally, we assessed lipase gene expression and found that H/R reduced the expression of both neutral lipases, adipose triglyceride lipase (ATGL), monoacylglycerol lipase (MGL), and hormone‐sensitive lipase (HSL), as well as the acidic lipase lysosomal acid lipase (LAL) (Figure 1H). Collectively, these results demonstrate that H/R reduces cellular LD content in cardiomyocytes, indicating altered LDs dynamics and metabolism under these conditions.

### 2.2 Lipophagy Contributes to LD Degradation Triggered by H/R

**FIGURE 2:**
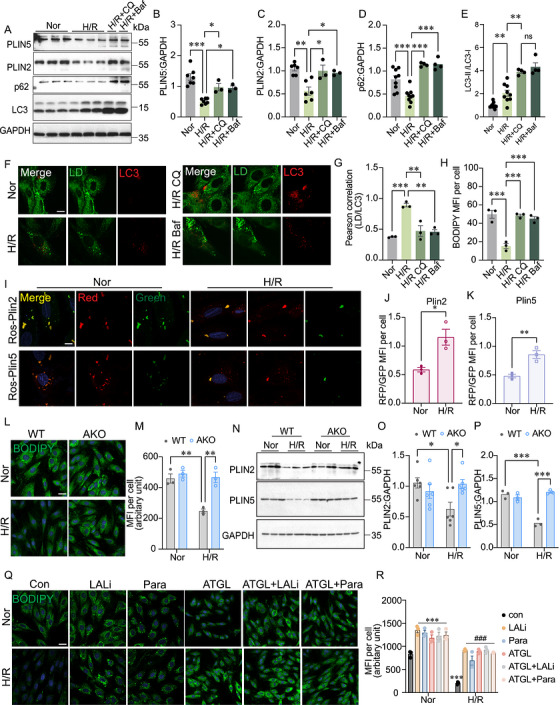
Lipophagy contributed to LD degradation during H/R in H9c2 cell. (A) SDS‐PAGE and immunoblot analysis of H9c2 lysate using PLIN5, PLIN2, LC3, p62, and GAPDH was used as a loading control. H9c2 cells were treated with or without 50 µM chloroquine (CQ), 50 µM bafilomycin (Baf), and subjected to normoxia or H/R. (B–E) Quantification of PLIN5, PLIN2, LC3, and p62. (F) Representative image of immunofluorescence staining of LC3 and BODIPY in H9c2 treated with or without 50 µM CQ, 50 µM Baf, and subjected to normoxia or H/R. (G and H) Pearson correlation of LC3 and BODIPY, and MFI (mean fluorescence intensity) quantification of BODIPY (F). (I) Representative image of Rosella PLIN2–H9c2 cell and of Rosella PLIN5–H9c2 cell subjected to normoxia or H/R, and quantification in (J) and (K). (L) Representative image of BODIPY staining in WT H9c2 cell and AKO cell subjected to normoxia or H/R, and quantification in (M). (N) SDS‐PAGE and immunoblot analysis of WT H9c2 cell (WT) and ATG7KO‐H9c2 cell (AKO) lysate using PLIN5, PLIN2, and GAPDH was used as a loading control. (O and P) Quantification of PLIN2 and PLIN5. (R) Representative image of BODIPY staining in H9c2 cell treated with or without 10 µM Lalistat 1 (LALi), 100 µM paraoxon, 20 µM atglistatin (ATGL), combination of LALi and ATGL, and combination of ATGL and paraoxon (Para) and subjected to normoxia or H/R, and quantification in (P). Scale bar: 10 µm. Results are presented as mean ± SEM (*n* = 3–6 per group). *p < 0.05, **p < 0.01, ***p < 0.001 versus normoxia control. ^###^ *p *< 0.001 versus H/R control. One‐way ANOVA was run for statistical analysis. Two‐way ANOVA was run when comparing groups on two different categorical variables.

In addition to the breakdown of TAG in LDs through cytosolic lipolysis, LDs can be engulfed by autophagosomes for lysosomal degradation, a process known as lipophagy [37, 38]. Pathway analysis revealed autophagy activation following H/R exposure (Figure 1E), prompting the investigation of lipophagy's role in H/R‐mediated LD catabolism. We detected diminished expression of PLIN2 and PLIN5 proteins after H/R treatment (Figure 2A–C), while autophagic flux simultaneously increased, as evidenced by elevated LC3‐II levels and enhanced p62 degradation (Figure 2A,D,E). Inhibition of lysosome acidification by chloroquine (CQ) or inhibition of autophagosome–lysosome fusion at preventing the acidification of the lysosomes by bafilomycin (Baf) disrupted autophagic flux (Figure S2A–C) and significantly mitigated the H/R‐associated decline in PLIN2 and PLIN5 levels (Figure 2A–E). Additionally, we observed increased colocalization of LC3 with LDs following H/R, evidenced by reduced BODIPY MFI and elevated LC3 expression, consistent with lipophagy activation (Figures 2F,G and S2D). Blocking autophagic flux with CQ and Baf reversed these H/R‐associated alterations in BODIPY and LC3 patterns (Figures 2F,G and S2D).

To determine which PLIN proteins facilitated lipophagy during H/R stress, immunofluorescence analysis assessed spatial associations between PLIN2/LC3 and PLIN5/LC3 pairs. While both PLIN2/LC3 and PLIN5/LC3 associations intensified during H/R conditions, the PLIN5–LC3 interaction predominated (Figure S2E–H). Normoxia and H/R conditions showed comparable PLIN2–PLIN5 colocalization (Figure S2E,H). Autophagy suppression with Baf confirmed PLIN5's involvement in H/R‐mediated lipophagy, as evidenced by increased LC3 and PLIN5 upon H/R following autophagy inhibition, although the colocalization of LC3 and PLIN5 sustained (Figure S2I–L). We then employed Rosella–PLIN2 H9c2 cells and Rosella–PLIN5 H9c2 cells to assess specific autophagic targeting of PLIN2‐ and PLIN5‐decorated LDs during H/R stress. This reporter system relies on pH‐dependent fluorescence properties: both GFP and RFP emit signals at neutral pH, but GFP fluorescence is selectively quenched in the acidic environment after autophagosome–lysosome fusion [39]. Tagging PLIN with this system enables precise tracking of cellular lipophagy flux as reported previously [40]. During H/R, the ratio of red versus green fluorescence was increased in Rosella–PLIN2 (Ros–PLIN2) and Rosella–PLIN5 (Ros–PLIN5), indicating that PLIN2–LDs and PLIN5–LDs are both degraded by autophagy (Figure 2I–K). To examine the functional significance of the autophagy machinery in H/R‐dependent LD metabolism, we generated an autophagy‐deficient H9c2 cell line by deleting ATG7 (autophagy related 7) via CRISPR–Cas9 technology as previously reported [41]. Unlike their wild‐type counterparts, ATG7 KO cells (AKO) maintained their LD content during H/R exposure, as assessed with BODIPY staining (Figure 2L,M) and the expression of PLIN2 and PLIN5 (Figure 2N–P).

To delineate the relative contributions of lipophagy and lipolysis to LD breakdown during H/R, we employed specific inhibitors targeting key hydrolytic enzymes: Lalistat 1 (acidic lipase inhibitor; LALi), paraoxon (neutral lipase inhibitor; Para), and atglistatin (ATGL inhibitor). BODIPY staining revealed that LD degradation was effectively abolished under H/R regardless of whether lipophagy, lipolysis, or both pathways were inhibited individually or in combination, suggesting that both lipophagy and lipolysis contribute to LD catabolism during H/R injury in cardiomyocytes (Figure 2Q,R). Interestingly, upon lipolysis and lipophagy inhibition during H/R, the restoration of PLIN5 reduction induced by H/R was only observed in the presence of LALi, suggesting that the H/R‐induced PLIN5 degradation relied mostly on lipophagy (Figure S2M,N). These findings collectively establish PLIN5‐associated lipophagy as a central mechanism in H/R‐induced LD catabolism.

### 2.3 LD–Mitochondria Tethering Is Disrupted During H/R

**FIGURE 3:**
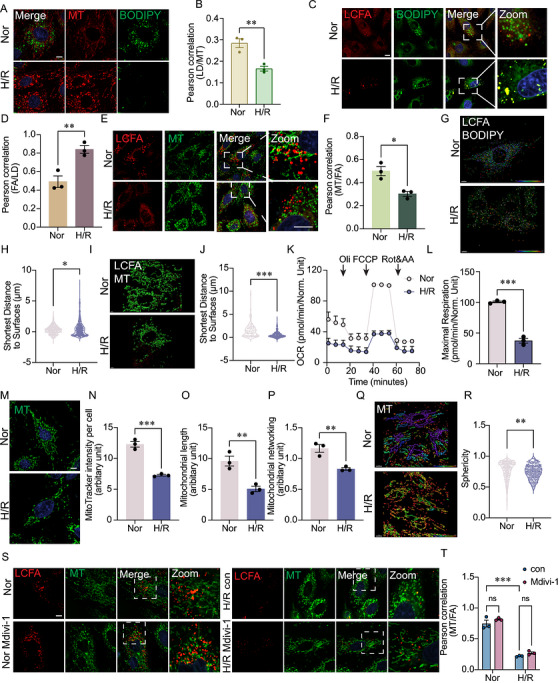
H/R disrupted LD and mitochondria tethering. (A) Representative image of Mitotrakcer (MT) and BODIPY (LD) staining in H9c2 cells subjected to normoxia or H/R. (B) Pearson correlation of MT and LD. (C) Representative image of long chain free fatty acid (LCFA) and BODIPY (LD) staining in H9c2 cells subjected to normoxia or H/R. (D) Pearson correlation of LCFA and LD. (E) Representative image of long chain free fatty acid (LCFA) and Mitotrakcer (MT) staining in H9c2 cells subjected to normoxia or H/R. (F) Pearson correlation of LCFA and MT. (G) Representative image of IMARIS analyzed long chain free fatty acid (LCFA) and BODIPY (LD) staining in H9c2 cells subjected to normoxia or H/R. (H) Quantification of (G). (I) Representative image of IMARIS analyzed long chain free fatty acid (LCFA) and Mitotrakcer (MT) staining in H9c2 cells subjected to normoxia or H/R. (J) Quantification of (I). (K) Seahorse mitochondrial stress test and quantification for parameters of mitochondrial function in H9c2 cells subjected to normoxia or H/R. (L) Quantification of maximal respiration capacity in Seahorse experiment. (M) Representative image of Mitotrakcer (MT) staining of H9c2 cells subjected to normoxia or H/R. (N–P) Quantification of mitochondria density, branch length, and networking. (Q) Representative image of IMARIS analyzed Mitotrakcer (MT) staining of H9c2 cells subjected to normoxia or H/R. (R) Quantification of sphericity of mitochondria. (S) Representative image of long chain free fatty acid (LCFA) and Mitotrakcer (MT) staining in H9c2 cells treated with or without 10 µM Mdivi‐1 and subjected to normoxia or H/R. (T) Pearson correlation of MT and LCFA. Scale bar: 10 µm. Results are presented as mean ±  SEM (*n* = 3 per group). *p < 0.05, **p < 0.01, ***p < 0.001 versus normoxia control. One‐way ANOVA was run for statistical analysis. Two‐way ANOVA was run when comparing groups on two different categorical variables.

LDs act as reservoirs of fatty acids, which are transported to mitochondria for β‐oxidation and ATP production under stress conditions or nutrient limitations [42]. PLIN5 is essential for LD–mitochondrial interactions and promoting fatty acid transport [18, 43, 44, 45]. Building on our findings that H/R enhances PLIN5–LD lipophagy, we next investigated whether H/R affects PLIN5‐mediated LD–mitochondria interactions. To assess this, we first examined the proximity of LDs and mitochondria during H/R. As expected, H/R disrupted the interaction of these two organelles, as evidenced by decreased proximity (Figure 3A,B). To further characterize this disruption, we employed a LCFA tracking assay, as previously reported [46], to trace the fate of lipids stored in LDs under H/R. Under normoxia, a substantial portion of LFCA localized outside LDs, indicating normal mobilization (Figure 3C,D). In contrast, under H/R conditions, LCFAs were predominantly colocalized with LDs (Figure 3C,D), while LCFA colocalization with mitochondria was markedly reduced (Figure 3E,F), suggesting a failure of LCFA transport to mitochondria for subsequent β‐oxidation. These findings were further validated using 3D IMARIS colocalization analysis (Figure 3G–J).

We next examined expression patterns of genes involved in fatty acid transport. The expression of *Cd36* and *Fabp4* was increased under H/R (Figure S3A,B), while the expression of mitochondrial fatty acid oxidation‐related genes, including *Cpt1α, Pparα*, and *Rxrα*, was downregulated (Figure S3C,D). *Fatp1* expression remained unchanged, whereas the lipophagy‐related gene Rab7 expression increased under H/R (Figure S3D,E). CPT1α protein expressed presented the same changes as the gene level upon H/R challenge (Figure S3F,G). Additionally, extracellular FFA levels were elevated upon H/R (Figure S3H), collectively suggesting that H/R does not impair intracellular or extracellular fatty acid transport but specifically reduces LCFA transport to mitochondria.

Given that efficient LD–mitochondria tethering facilitates ATP production via β‐oxidation, we assessed cellular bioenergetics using the Seahorse extracellular flux analysis. Seahorse extracellular flux analysis revealed that H/R drastically reduced maximal respiratory capacity in H9c2 cells compared with normoxia control (Figure 3K,L). Basal respiration and ATP production were similarly diminished (Figure S3I), consistent with impaired fatty acid oxidation and mitochondrial dysfunction. Under normoxia, pharmacological autophagy inhibition by CQ, or genetic ablation of ATG7 (ATG7 KO) reduced maximal respiration versus WT, indicating basal autophagic activity supports mitochondrial function. However, during H/R, neither CQ nor ATG7 KO exacerbated respiratory suppression, suggesting that H/R itself or H/R‐induced loss of LD–mitochondria contacts overwhelms autophagy‐dependent metabolic regulation (Figure S3J,K).

To investigate mitochondrial structural changes under H/R, we analyzed mitochondrial morphology. H/R decreased mitochondrial density, length, and networking (Figure 3M–Q). IMARIS sphericity analysis, used as the index of fission, revealed increased mitochondrial fragmentation during H/R (Figure 3R,S). This fragmentation was further corroborated by increased p‐DRP1 (Ser 616), decreased p‐DRP1 (Ser 637), and elevated MFF, and decreased MFN2 levels (Figure S4A–F). Additionally, H/R significantly decreased *Pparα* and *Rxrα* mRNA expression in H9c2 (Figure S4G,H). Concurrently, H/R stimulated mitophagy as evidenced by increased Parkin, and LC3 expression and decreased p62 in mitochondrial fraction (Figure S4I–L), together suggesting that decreased nuclear receptor activity contributes to enhanced mitochondrial degradation during H/R‐induced stress in cardiomyocytes.

To examine whether H/R‐induced mitochondrial fission contributed to impaired fatty acid utilization, we treated cells with the mitochondrial fission inhibitor Midivi‐1. This intervention successfully alleviated fission by measuring mitochondrial morphology (Figure S4M–P). Interestingly, despite preserving mitochondrial morphology induced by H/R, LCFA remained sequestered in LDs and failed to transfer to mitochondria for utilization (Figure 3S,T). This observation suggests that mitochondrial fragmentation is not the primary factor responsible for disrupted LD–mitochondrial tethering and subsequent impairment of fatty acid metabolism during H/R.

### 2.4 Identification of Rab8a as a Critical Regulator of LD–Mitochondria Fatty Acid Transfer in H9c2 Cells During H/R

**FIGURE 4:**
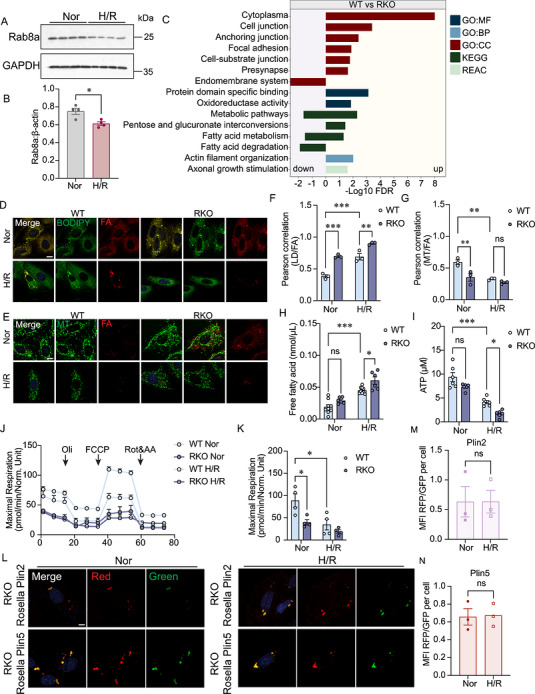
Rab8a role in the LD and mitochondrial tethering during H/R. (A) SDS‐PAGE and immunoblot analysis of H9c2 lysate using Rab8a, and GAPDH was used as loading control. (B) Quantification of Rab8a. (C) Pathway enrichments of significant expressed proteins. (D) Representative image of long chain free fatty acid (LCFA) and BODIPY (LD) staining in WT and Rab8a KO H9c2 cell (RKO) subjected to normoxia or H/R. (E) Representative image of long chain free fatty acid (LCFA) and Mitotrakcer (MT) staining in WT and RKO subjected to normoxia or H/R. (F and G) Pearson correlation of FA and LD, FA and MT. (H) Extracellular free fatty acid measurement in culture medium of WT and RKO subjected to normoxia or H/R. (I) ATP measurement in culture medium of WT and RKO subjected to normoxia or H/R. (J) Seahorse mitochondrial stress test and quantification for parameters of mitochondrial function in WT and RKO subjected to normoxia or H/R. *Abbreviations*: Oil: oligomycin; FCCP: carbonyl cyanide‐p‐(trifluoromethoxy) phenylhydrazone; Rot: rotenone; AA: antimycin. (K) Quantification of maximal respiration capacity in Seahorse experiment. (L) Representative image of Rosella PLIN2–RKO and of Rosella PLIN5–RKO subjected to normoxia or H/R and quantification in (M) and (N). Scale bar: 10 µm. Results are presented as mean ± SEM (*n* = 3–4) per group. *p < 0.05, **p < 0.01, ***p < 0.001 versus normoxia control. One‐way ANOVA was run for statistical analysis. Two‐way ANOVA was run when comparing groups on two different categorical variables.

Rab small GTPases serve as key regulators of membrane dynamics, including trafficking, contact formation, and fusion [47]. Among Rab family proteins, Rab8a has been identified as a mitochondrial receptor for LDs in skeletal muscle [20] and mediates LD fusion in adipocytes [22]. However, its role in cardiomyocytes remains uncharacterized. Notably, we observed significant downregulation of Rab8a protein expression in H9c2 cells subjected to H/R (Figure 4A,B). To investigate the functional significance of Rab8a during H/R, we generated Rab8a knockout H9c2 cells (RKO) using CRISPR‐Cas9 technology. Knockout efficiency was confirmed by western blot analysis (Figure S5A,B). Proteomic analysis was performed to measure the functional significance of Rab8a. Hierarchical clustering heatmaps and PCA revealed distinct proteome profiles between WT and RKO (Figure S5C,D). Pathway enrichment analysis comparing RKO and WT indicated that fatty acid degradation and fatty acid metabolism were significantly dampened when Rab8a was deleted (Figure 4C). We then evaluated LD–mitochondria interactions under various conditions. Under normoxic conditions, Rab8a deletion significantly increased the spatial separation between these organelles, an effect that was further amplified during H/R exposure (Figure S5E,F). Next, we assessed LCFA trafficking in RKO versus WT cells under both normoxia and H/R conditions. Rab8a deficiency significantly impaired LCFA mobilization from LDs, as evidenced by an increased overlap coefficient between LCFA and LDs under normoxia, which was further exacerbated during H/R (Figures S5G,H and 4D,F). However, Rab8a deficiency did not further exacerbate the H/R‐induced diminution of LCFA overlapping coefficient with mitochondria (Figures S5G,I and 4D,G). Intracellular and extracellular FFA levels were unchanged between WT and RKO cells (Figures 4H and S5J,K). Moreover, we validated the protein interaction of Rab8a with Tom20 and PLIN5 upon H/R by immunoprecipitation. We found that there under normoxic conditions, Tom20 and PLIn5 was both expressed in H9c2 cell lysate immunoprecipitated with Rab8a antibody conjugated with protein A/G agarose beads, however, decreased under H/R condition (Figure S5L,M). Functionally, RKO cells exhibited a marked reduction in ATP production compared with WT cells during H/R (Figure 4I). The mitochondrial respiratory impairment was further substantiated by Seahorse extracellular flux analyses, which revealed significantly impaired mitochondrial respiration in Rab8a‐deficient cells (Figure 4J,K). To determine whether Rab8a influences H/R‐induced PLIN‐mediated lipophagy, we expressed Rosella‐PLIN2 and Rosella–PLIN5 in RKO cells. In contrast to WT cells (Figure 1I–K), no significant increase in PLIN2‐ or PLIN5‐associated lipophagy was observed upon H/R in RKO cells (Figure 4L–N). Collectively, these data establish Rab8a as a critical mediator of LD–mitochondria LCFA transfer that becomes dysregulated during H/R, contributing to impaired bioenergetics and cellular stress in cardiomyocytes.

### 2.5 ALY688 Restores LD–Mitochondria Communication by Replenishing Rab8a Activation Upon H/R and Protects Against Cell Death

**FIGURE 5:**
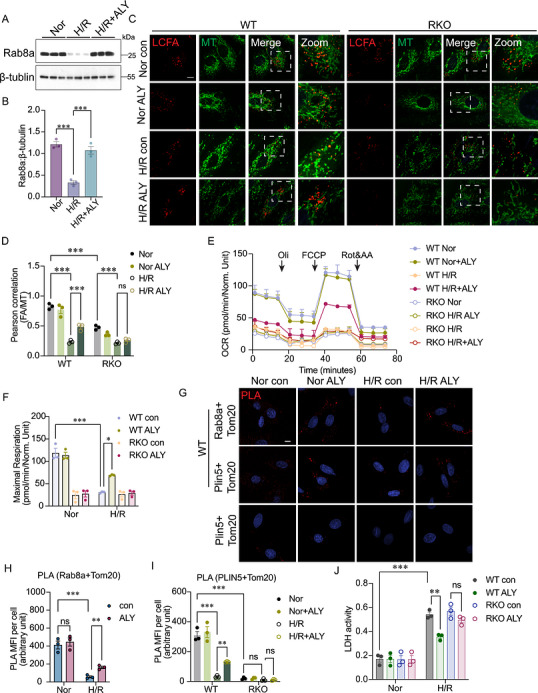
ALY688 improved LD and mitochondria communication during H/R. (A) SDS‐PAGE and immunoblot analysis of H9c2 lysate using Rab8a, and β‐tubulin was used as loading control. (B) Quantification of Rab8a. (C) Representative image of long chain free fatty acid (LCFA) and Mitotracker (MT) staining in H9c2 treated with or without 100 nM ALY688 (ALY) and subjected to normoxia or H/R. (D) Pearson correlation of FA and MT. (E) Seahorse mitochondrial stress test and quantification for parameters of mitochondrial function in H9c2 treated with or without 100 nM ALY688 and subjected to normoxia or H/R. *Abbreviations*: Oil: oligomycin; FCCP: carbonyl cyanide‐p‐(trifluoromethoxy) phenylhydrazone; Rot: rotenone; AA: antimycin. (F) Quantification of maximal respiration capacity in Seahorse experiment. (G) Representative image of PLA (proximity ligation assay) using the combination of Rab8a and Tom20, PLIN5 and Tom20 in WT and RKO treated with or without 100 nM and subjected to normoxia or H/R. (H) Quantification of Rab8a and Tom20 PLA MFI. (I) Quantification of PLIN5 and Tom20 PLA MFI. (J) LDH measurement of WT, RKO, treated with or without ALY688, and subjected to normoxia or H/R. Scale bar: 10 µm. Results are presented as mean ± SEM (*n* = 3) per group. *p < 0.05, **p < 0.01, ***p < 0.001 versus normoxia control. One‐way ANOVA was run for statistical analysis. Two‐way ANOVA was run when comparing groups on two different categorical variables.

Previous studies have demonstrated that AMPK activation enhances LD motility and promotes LD–mitochondria interactions to facilitate fatty acid oxidation [48]. However, whether adiponectin, a well‐established AMPK activator, regulates LD–mitochondria interaction in cardiomyocytes remains unclear. Here, we investigated this using the adiponectin receptor agonist ALY688. As reported previously 30 min pretreatment with ALY688 activated adiponectin signaling pathway [49]. In this study, we pretreated ALY688 30 min prior to H/R challenge. As shown, ALY688 significantly prevented the H/R‐induced reduction of Rab8a protein expression (Figure 5A,B), suggesting a potential regulatory relationship between adiponectin signaling and Rab8a‐mediated organelle tethering. Besides, when AMPK inhibitor Compound C was used, the reactivation of Rab8a upon ALY688 treatment was diminished, indicating the effect derived from ALY688 was potentially dependent on AMPK pathway (Figure S6A,B). To assess the functional impact of ALY688 on fatty acid trafficking, we performed an LCFA tracking assay. Consistent with enhanced LD–mitochondria coupling, ALY688 treatment significantly stimulated LCFA mobilization from LDs to mitochondria during H/R (Figure 5C,D). Additionally, the H/R induced decreased Rab8a–Tom20 and Rab8a–PLIN5 interaction were improved upon ALY688 treatment, as determined by immunoprecipitation (Figure S6C). ALY688‐mediated restoration of LD–mitochondria proximity significantly attenuated H/R‐induced impairment of fatty acid oxidation, as evidenced by partial recovery of mitochondrial β‐oxidation capacity (Figure S6D,E). Importantly, this beneficial effect on LD–mitochondria proximity was completely abolished in Rab8a‐deficient cells (Figures 5C,D and S6F,G), demonstrating that Rab8a is essential for mediating adiponectin's effects on intracellular lipid trafficking. These findings were further validated using Seahorse extracellular flux analysis, which confirmed that ALY688‐induced improvements in mitochondrial respiration during H/R required Rab8a expression (Figure 5E,F). To further validate these findings, we used a proximity ligation assay, a sensitive in situ method for inferring protein‐protein interactions with high specificity [50]. In WT cells, H/R reduced the association of Rab8a with the mitochondrial marker Tom20, as well as PLIN5 and Tom20 (Figure 5G–I). ALY688 treatment effectively restored these interactions during H/R. Notably, in Rab8a knockout cells, the ability of ALY688 to restore organelle contacts was completely abrogated (Figure 5I), further confirming Rab8a's indispensable role in mediating adiponectin‐induced organelle tethering. To evaluate the functional significance of ALY688‐mediated Rab8a‐dependent LD–mitochondria coupling during H/R, we measured the cell viability using an LDH release assay. ALY688 pretreatment significantly reduced H/R‐induced cell death in WT cells (Figure 5J). However, this cytoprotective effect was completely abolished in Rab8a‐deficient cells (Figure 5J), demonstrating that Rab8a is required for the protective effects of adiponectin receptor activation during H/R injury. Collectively, these findings establish that Rab8a‐mediated interactions between LDs and mitochondria are critical for maintaining efficient fatty acid oxidation and cardiomyocyte viability during metabolic stress. Furthermore, they identify the adiponectin–Rab8a axis as a novel therapeutic target for protecting cardiomyocytes against H/R‐induced metabolic dysfunction and cell death.

## 3 Discussion

Cardiac energy metabolism depends on the precise coordination of lipid storage, mobilization, and mitochondrial oxidation. These processes are severely disrupted during ischemic stress. IR injury affects cardiac energy metabolism through multiple mechanisms. Among them, impaired lipid handling is a key contributor to cardiomyocyte damage [51]. Physical and functional coupling between LDs and mitochondria are essential for maintaining metabolic flexibility and preventing lipotoxicity in the heart [4, 19, 52, 53]. This study uncovers a previously unrecognized mechanism of metabolic dysfunction during H/R. Rab8a‐mediated LD–mitochondria tethering is downregulated, impairing fatty acid utilization and promoting lipotoxicity. We found that the adiponectin receptor agonist ALY688 provides cardioprotection partly by restoring this communication axis. These findings enhance our understanding of ischemic injury and suggest potential therapeutic strategies.

MCSs serve as crucial metabolic junctions for intracellular trafficking and signaling. We demonstrate that H/R significantly disrupted LD–mitochondria proximity, compromising fatty acid channeling despite concurrent activation of lipid mobilization pathways. This uncoupling represents a fundamental aspect of metabolic inflexibility during cardiac stress. Recent ultrastructural analyses have established that approximately 30–45% of LDs in healthy cardiomyocytes maintain close proximity (<100 nm) to mitochondria, facilitating efficient substrate delivery [52]. The disruption of these contacts during H/R, coupled with downregulation of fatty acid oxidation genes (*CPT1α*, *PPARα*, *RXRα*), creates a severe mismatch between lipid mobilization and utilization that contributes to accumulation of lipotoxic intermediates, particularly DAG, as documented in various models of cardiac stress [2, 9]. It is worth noting that the relationship between cardiac lipid metabolism and hypoxic signaling is likely bidirectional. Recent evidence suggests that defects in lipid handling can induce a state of “pseudo‐hypoxia,” where metabolic intermediates or oxidative stress stabilize HIF‐1α even in the absence of severe environmental hypoxia [54, 55, 56, 57, 58]. In our study, the HR‐induced loss of Rab8a leads to an accumulation of untethered LDs and FFAs. The subsequent lipotoxicity and generation of ROS from these accumulated lipids may inhibit prolyl hydroxylases, thereby reinforcing HIF‐1α stabilization. This lipid‐driven pseudo‐hypoxic state could potentially create a maladaptive feedback loop, further entrenching the metabolic inflexibility observed in ischemic cardiomyocytes.

Here, we show that lipid catabolism during H/R occurs through both lipolysis and lipophagy, the latter being particularly significant for predominant degradation of PLIN5‐coated LDs. While lipophagy has been extensively studied in hepatocytes and adipocytes [13, 16, 24, 37, 59, 60], its role in cardiomyocyte metabolism during ischemic stress has remained less defined. The observation that inhibition of LAL specifically prevents PLIN5 degradation during H/R highlights selective vulnerability of PLIN5‐coated LDs to autophagy‐mediated degradation. This selectivity likely reflects specific cargo receptor interactions that have been described in recent studies of selective autophagy [61, 62, 63]. Lipophagy generally serves as an adaptive response to provide substrates for mitochondrial oxidation during nutrient deprivation [64, 65]. However, when LD–mitochondria coupling is disrupted, as seen with reduced Rab8a activity during H/R, enhanced lipophagy appears to become metabolically uncoupled. While lipophagy typically supports energy production, in the absence of Rab8a‐mediated tethering, the fatty acids mobilized by autophagy cannot be efficiently transferred to mitochondria. This mismatch between lipid mobilization and oxidation likely results in the accumulation of lipotoxic intermediates rather than ATP synthesis.

The identification of Rab8a as a key regulator of LD–mitochondria tethering in cardiomyocytes expands its known roles beyond vesicular trafficking to include metabolic coordination. Rab8a has also been previously linked to LD fusion in adipocytes [22] and identified as a mitochondrial receptor for LDs in skeletal muscle [20], its role in cardiac lipid metabolism remained unexplored until now. Rab8a deficiency mirrors key features of H/R‐induced metabolic dysfunction. It increases LD–mitochondria proximity, impairs LCFA mobilization, and reduces bioenergetic capacity. The fact that Rab8a does not influence PLIN5‐associated lipophagy suggests it specifically mediates organelle tethering rather than lipid breakdown, highlighting its specialized role in metabolic coordination. Recent super‐resolution microscopy studies show extensive contact networks between organelles, with about 90% of LDs maintaining stable contacts with at least one other organelle [66]. H/R‐induced Rab8a downregulation disrupts this intricate communication network, with profound consequences for metabolic homeostasis.

Preventing mitochondrial fragmentation using the inhibitor Mdivi‐1 did not restore LCFA transfer to mitochondria during H/R in cardiomyocytes. This observation challenges the prevailing paradigm that mitochondrial structural integrity directly determines metabolic function. Our data suggest that LD–mitochondria tethering rather than mitochondrial morphology itself constitutes the critical determinant of efficient fatty acid trafficking. This distinction carries implications for therapeutic strategies targeting mitochondrial dysfunction in cardiac disease. Recent investigations have demonstrated that mitochondrial fission and fusion proteins participate in tethering mitochondria to other organelles, suggesting that their distinct roles extend beyond morphology regulation to include interorganelle communication [18, 19, 23, 43, 44, 67, 68, 69, 70]. These findings align with emerging research showing that mitochondrial dynamics and substrate preference can be mechanistically uncoupled under specific conditions, supporting the broader conclusion that organelle tethering represents a distinct regulatory layer in cardiac metabolism [71, 72, 73].

ALY688 restores Rab8a expression and LD–mitochondria tethering, offering both mechanistic insight and therapeutic potential. In Rab8a‐KO cells, ALY688's beneficial effects are completely lost, confirming Rab8a as a key mediator of adiponectin's metabolic actions. Adiponectin itself has been widely studied for cardioprotection during IR, with benefits linked to AMPK activation, anti‐inflammatory effects, and enhanced mitochondrial function [74]. These findings add a new dimension to adiponectin's protective effects by showing its role in preserving organelle communication via Rab8a. Mechanistically, this likely involves AMPK‐dependent activation of Rab8a. Recent studies show that AMPK phosphorylation increases GTP‐bound active Rab8a, which promotes LD–mitochondria tethering through binding to PLIN5 [20]. These results establish a direct link between adiponectin signaling, organelle tethering, and cardiomyocyte survival during metabolic stress.

Targeting the adiponectin–Rab8a axis could help restore metabolic flexibility by improving fatty acid utilization. The fact that ALY688 reduces cell death during H/R indicates that preserving LD–mitochondria tethering may protect against lipotoxicity‐induced apoptosis, a major contributor to ischemic injury and heart failure progression [7, 8, 18]. This mechanism complements adiponectin's established effects on cardiac remodeling, inflammation, and oxidative stress. Together, these actions may provide a multi‐faceted approach to cardioprotection.

In conclusion, this study identifies Rab8a‐mediated LD–mitochondria tethering as a critical metabolic junction that becomes dysregulated during H/R in cardiomyocytes. The findings demonstrate that the adiponectin receptor agonist ALY688 restores this communication axis and attenuates metabolic dysfunction, providing mechanistic insight into adiponectin's cardioprotective actions and identifying Rab8a as a promising therapeutic target for ischemic heart disease.

### 3.1| Limitations

While our data show that Rab8a deletion exacerbates HR injury and ALY688 mitigates it, we did not perform direct Rab8a re‐expression experiments in the knockout background. Therefore, we cannot rule out that ALY688 may exert protective effects through parallel, Rab8a‐independent pathways. Future studies involving genetic rescue of Rab8a will be required to definitively establish its sufficiency in preventing HR‐induced metabolic dysfunction. Additionally, while we infer that the increased lipophagic flux is detrimental based on the concurrent reduction in ATP and accumulation of lipid intermediates, we did not normalize autophagic flux directly to mitochondrial β‐oxidation rates in real‐time. Future studies using dual‐pulse chase assays or flux–metabolism coupling models would be beneficial to quantitatively define the “efficiency ratio” of lipophagy‐derived fuel utilization under stress conditions.

## 4 Methods and Materials

### 4.1 Cell Culture and Cell Line Generation

H9c2 rat embryonic cardiac myoblasts (ATCC CRL‐1446) were cultured in Dulbecco's modified Eagle's medium (Gibco; 11885‐084) with 10% fetal bovine serum (Thermo; 11885084) and 1% streptomycin/penicillin (vol/vol; Gibco; 15070063) at 37°C under 5% CO_2_. Hypoxia was induced by placing cells in a hypoxic chamber (Onstage Incubator; Thermo Fisher, NX7LIVE001) containing a premixed gas of 1% O_2_ and 5% CO_2_. Normoxia and reoxygenation were achieved by incubating cells in the same chamber with 20% O_2_ and 5% CO_2_.

CRISPR knock‐out cell lines were created using guide RNAs cloned into the pX459 plasmid, as previously described [75]. H9c2 cells were transfected with vectors carrying guides targeting atg7 using Lipofectamine 3000. Transduced cells were selected with 1 µg/mL puromycin for 5 days and then expanded in media without selection. T7 endonuclease assays were performed using T7 endonuclease I on PCR products generated with the primers 5′‐GCTGCTGCAGGTAGGTGTAA and 5′‐GGTGTCCTGTCTGAGACTGC, following the manufacturer's instructions.

To generate Rab8a KO H9c2 cells, guide RNA targeting Rab8a gene (TGATTGTCCGAAACCGCTCC) are cloned in pLentiCRISPRv2. We then generated viral particles using this vector as well as a control vector that does not target mammalian genomes and used these particles to transduce H9c2 cells. Following selection with puromycin (1 µg/mL), polyclonal populations were assessed for guide efficiency using a T7 endonuclease I assay as reported before [75].

Lentiviruses for stable expression of Rosella–Plin2 (rat) or Rosella–Plin5 (rat) were produced at the University of Ottawa Genomic Editing and Molecular Biology (GEMb) core facility. H9c2 cells were transduced with these lentiviruses in the presence of 8 µg/mL polybrene (Sigma; H9268). Transduced cells were then selected by fluorescence‐activated cell sorting using a BD FACS Aria IIIu (Beckton Dickinson).

### 4.2 Western Blot

Cells were lysed in RIPA buffer containing protease and phosphatase inhibitors. Protein concentration was measured and normalized using a BCA assay (Thermo; A55864). Proteins were separated by reducing SDS‐PAGE, transferred to PVDF membranes, and incubated with primary antibodies overnight at 4°C. Secondary antibodies were applied for 1 h at room temperature. Signal quantification was performed by densitometry of scanned autoradiographs using ImageJ (version 1.4v).

### 4.3 Immunoprecipitation

Lysates were prepared from H9c2 cells in ice‐cold lysis buffer containing 20 mM Tris–HCl pH 8, 137 mM NaCl, 10% glycerol, 1% NP‐40, 2 mM EDTA, Complete Protease Inhibitor Cocktail. Cell lysates were then immunoprecipitated with Rab8a antibody conjugated with protein A/G agarose beads (ThermoFisher; 20421) and then resolved on 12 SDS gel before being probed with indicated antibodies. IgG was used as isotype control (Proteintech; 98136‐1‐RR).

### 4.4 Inhibitors, Chemicals, and Antibodies

The following chemicals, dyes, and antibodies were used: 100 nM rapamycin (Sigma; R8781), 50 µM CQ (Sigma; C6628), 50 µM Baf (Sigma; 196000), 100 µM Para (Sigma; 36186), 10 µM Lalistat 1 (Med Chem Express; HY‐116815), 60 µM etomoxir (Sigma; 236020),1 µM MDIVI‐1 (Med Chem Express; HY‐15886), 20 µM Compound C (Sigma; 171260), 100 nM ALY688 (Allysta Pharmaceutical), 1 mM BODIPY 558/568 C12 (Thermo; D3835), 10 mM BODIPY 493/503 (Thermo; D3922), 100 nM MitoTracker Red (Thermo; M22425), 100 nM MitoTracker Green (Thermo; M7514), 300 nM Hoechst (Thermo; H10325), GAPDH (Thermo Fisher; MA5‐15738), β‐tubulin (Cell Signaling; 2128), Rab8a (BD Science; 610844), PLIN2 (Proteintech; 60340), PLIN5 (Proteintech; 26951), LC3 (Cell Signaling; 2775), p62 (Cell Signaling; 5114), HIF1⍺ (Cell Signaling; 79233), CPT1⍺ (Proteintech; 15184‐1‐AP), Tom20 (Proteintech; 66777), VDAC (Cell Signaling; 4661), P‐DRP1 Ser616 (Cell Signaling; 3455), P‐DRP1 Ser637 (Cell Signaling; 4867), Parkin (Proteintech; 66674), MFF (Cell Signaling; 84580), Mitofusin 2 (Cell Signaling; 9482), anti‐rabbit (Cell Signaling; 7074), and anti‐mouse (Cell Signaling; 7076).

### 4.5 Immunofluorescence

Cells were washed three times with 1× PBS and fixed with 4% paraformaldehyde (Sigma; HT501128) for 10 min at room temperature. Cells were then blocked and permeabilized for 30 min in 5% BSA with 0.01% Triton X‐100. Primary antibodies were applied overnight at 4°C in wash buffer containing 1% BSA and 0.025% saponin. Alexa Fluor or Alexa Fluor Plus‐conjugated secondary antibodies were incubated for 1 h at 37°C in the same wash buffer. Cells were counterstained with DAPI for 5 min at room temperature. Slides were imaged using a Nikon Eclipse Ti2 microscope for visualization and continuous measurements.

### 4.6 Image Analysis and Quantification

Image analysis was performed using ImageJ/Fiji (NIH) and Imaris software (Bitplane). For LD quantification, the MFI of BODIPY staining was measured per cell using ImageJ, with background subtraction applied prior to quantification. LD number and size were analyzed using the “Analyze Particles” plugin in ImageJ after thresholding.

Colocalization analysis was conducted using the Imaris Coloc module on 3D‐reconstructed Z‐stack images. The degree of overlap between channels (e.g., LDs and mitochondria, or LC3 and LDs) was quantified using Mander's overlap coefficient or Pearson's correlation coefficient, as indicated in the figure legends. For mitochondrial morphology assessment, 3D surface rendering was performed in IMARIS to determine sphericity (as an index of fragmentation), while mitochondrial length and network density were analyzed using the IMARIS Filament Tracer tool. All analyses were performed on at least 30 cells per condition across three independent biological replicates.

### 4.7 Quantitative Real‐Time PCR

Total RNA was extracted using the PureLink RNA Mini Kit (Thermo; 12183018A), and first‐strand cDNA was synthesized with the cDNA Synthesis Kit (Thermo; K1691) following the manufacturer's instructions. qPCR was performed using SYBR Green Supermix (Bio‐Rad; 1725274). Sequence of primers are listed in Table S1. Data were normalized to 18S RNA, and fold changes were calculated using the 2^−ΔΔCt^ method. Relative mRNA levels are expressed in arbitrary units, with the untreated group set to 1.

### 4.8 Mitochondrial Isolation

Muscle samples were homogenized with a glass‐Teflon Dounce Homogenizer in extraction buffer (70 mM sucrose, 1 mM EGTA, 10 mM mannitol, 5 mM HEPES, 10 mM MgCl_2_, protease, and phosphatase inhibitor cocktail). The homogenates were centrifuged twice at 1000×*g* for 10 min to remove the tissue debris and nucleus. The post nuclear supernatants were then transferred into 1.5 mL Eppendorf tubes and adjusted to equal concentrations. Equal amounts of PNS were centrifuged at 10,000×*g* for 10 min to precipitate mitochondria. The pellets were washed in extraction buffer two times with centrifugation at 10,000×*g* for 10 min in between. The mitochondrial fractions were dissolved in SDS sample buffer for immunoblotting analysis.

### 4.9 Biochemistry Analysis

Commercially available kits were used to measure LDH (Abcam; ab102526), FFA (Abcam; ab65341), ATP (Abcam; GA5010), according to manufacturer's instructions.

### 4.10 Seahorse Flux Assay

Oxygen consumption rates were determined by a Seahorse XFe24 extracellular flux analyzer (Agilent) as previously described [76]. Briefly, WT H9c2 cells, RKO, and AKO were seeded in each well of a precoated XFe24 cell culture plate (Agilent: 103015) and treated with Lalistat 1, Para, ATGL, rapamycin, Baf, and ALY688 prior H/R. After H/R, cells were washed with XF media twice and incubated in a CO_2_‐free incubator at 37°C for 1 h. The following reagents were used: oligomycin (1 µM; Sigma; 75351), FCCP (carbonyl cyanide p‐trifluoromethoxy phenylhydrazone, 3 µM; Sigma; C2920), and antimycin A (1 µM; Sigma; A8674) plus rotenone (1 µM; Sigma; R8875).

### 4.11 Lipidomics Analysis

The lipids from H9c2 cells and whole heart tissues were extracted in (1:10 w:v mg:uL) extraction solvent butanol:MeOH (1:1) containing 1 µg/mL Equisplash internal standard mix. The samples were homogenized, sonicated, and centrifuged 13,000x*g* for 10 min at room temperature (23°C). ∼100 µL of each sample supernatants were used for the LC–MS analysis. Leftover samples were transferred to −20°C storage. Pooled samples for each group were made by combining 5 µL of each sample (Total). LC–MRM–MS analyses were performed using Agilent 1290 LC with 16 min gradient (A) 0.1% formate in 5:3:2 water:acetonitrile:2‐propanol. (B) 0.1% formate in 1:9:90 water:acetonitrile:2‐propanol at 400 µL/min flow rate on Zorbax eclipse plus C18 RRHD 100×2.1 mm 1.8u (RP–LC–MS). 1 µL injection volumes were used for each run. MRMs were acquired using Agilent 6495A spectrometer in positive and negative modes in two separate runs. 519 and 768 lipid species were detected in H9c2 cells and heart tissues, respectively. Data for each LC/acquisition type were analyzed using MassHunter (Agilent). Statistical analysis was performed using MetaboAnalyst.

### 4.12 Proteomic Analysis

Isolated EVs were lysed in 5% SDS (Fisher Chemical), 200 mM TRIS (Fisher BioReagnets) pH 8.5 with 10 mM TCEP (60C x 25 min). Free disulfides were alkylated with 40 mM IAA (RT, dark, 30 min) prior to proteolytic digestion by S‐TRAP micro (see attached for the SOP from the vendor that we use). Peptide containing eluates were lyophilized and rehydrated at 10 ng/µL prior to loading on EvoTips and analysis by library free DIA using an Evosep One system coupled to a Bruker timsTOF HT MS operated in DIA‐PASEF mode. Samples were separated on an EV1137 column at 30SPD. For recipient cell proteomics, wild‐type L6 cells were seeded on six‐well plate until reaching 80–90% confluency. Confluent cells were treated with EVs isolated from mice plasma, at the concentration of 10 µg/mL. After 24 h of EVs treatment, cells were trypsinized and centrifuged for 5 min at 350 g. Cell pellets were collected and lysed in NP40 lysis buffer for 30 min on ice. Cell lysate was then subjected to in‐solution digestion following protocol by Promega (V5280). Gene ontology enrichment analysis was performed following the guidelines from the protocol using Cytoscape software (version 3.10.2). KEGG pathway enrichment analysis was performed using the ClueGO plugin (version 2.5.10) in Cytoscape software. ClueGO was used to identify significantly enriched KEGG pathways, applying two‐sided hypergeometric test for pathway analysis. The *p* values were adjusted for multiple testing using either the Bonferroni or Benjamini–Hochberg correction, with an adjusted *p* value threshold of 0.05.

### 4.13 Statistical Analysis

Data were presented as mean ± SEM. Statistical analysis was performed using GraphPad Prism 9. Student's *t*‐test was used for comparison of two groups and one‐way ANOVA or two‐way ANOVA were used for comparison of more than two groups. *p *< 0.05 was considered statistically significant.

### 4.14 Primer Sequences

The sequences of the primers used for qPCR analysis are provided in Table S1.

## Author Contributions

*Conceptualization*: Jialing Tang, Mireille Ouimet, and Gary Sweeney. *Methodology*: Jialing Tang, Nathan Joyce, Thomas Laval, Hye Kyoung Sung, Mireille Ouimet, and Gary Sweeney. *Investigation*: Jialing Tang, Nathan Joyce, Thomas Laval, and Eddie Tam. *Funding acquisition*: Mireille Ouimet and Gary Sweeney. *Projection administration*: Mireille Ouimet and Gary Sweeney. *Supervision*: Gary Sweeney. *Writing – original draft*: Jialing Tang. *Writing – review and editing*: Jialing Tang, Nathan Joyce, Thomas Laval, Mireille Ouimet, and Gary Sweeney. All authors have read and approved the final manuscript.

## Funding Statement

This work was supported by the Canadian Institutes of Health Research (CIHR; PJT‐391187), a Canada Research Chair awarded to M.O., and the Heart and Stroke Foundation of Canada (M.O.).

## Ethics Statement

The authors have nothing to report.

## Consent

The authors have nothing to report.

## Conflicts of Interest

GS acted as a consultant for Allysta Pharmaceuticals who provided ALY688. All other authors confirm no conflict of interest with the contents of this study.

## Supporting information




**Table S1**: Sequences of primers.
**Figure S1**: H/R altered ceramide species, and remodels lipid droplet dynamics and lipid metabolism gene expression in H9c2 cells. (A) SDS‐PAGE and immunoblot analysis of H9c2 lysate using HIF1⍺, β‐tubulin was used as a loading control. (B) Quantification of HIF1⍺. (C) Intensity of Cer (d18:1/16:0). (D) Intensity of Cer (d18:1/18:0). (E) Intensity of Cer (d18:1/24:1). (F) Quantification of number of LD of H9c2 cells subjected to normoxia or H/R. (G) Quantification of size of LD of H9c2 cells subjected to normoxia or H/R. (H–K) mRNA expression of PLIN1, PLIN3, PLIN4, DGAT1 in H9c2 cells subjected to normoxia or H/R. Results are presented as mean ± SEM (*n* = 3–9 per group). *p < 0.05, **p < 0.01, ***p < 0.001 versus normoxia control. One‐way ANOVA was run for statistical analysis.
**Figure S2**: H/R promoted lipid droplet‐associated autophagy and PLIN–LC3 interactions in H9c2 cells, modulated by lysosomal and lipolytic pathways. (A) SDS‐PAGE and immunoblot analysis of H9c2 lysate using p62 and LC3, GAPDH was used as a loading control. H9c2 cells were treated with or without 50 µM Chloroquine (CQ), and subjected to normoxia or H/R. (B and C) Quantification of p62 and LC3. (D) MFI (mean fluorescence intensity) quantification of LC3. (E) Representative image of immunofluorescence staining of LC3 and PLIN2 and PLIN5 in H9c2 subjected to normoxia or H/R. (F) Pearson correlation of PLIN2 and LC3, PLIN5, and LC3. (G) MFI quantification of PLIN2 and PLIN5. (H) Pearson correlation of PLIN2 and PLIN5. (I) Representative image of immunofluorescence staining of PLIN5 and LC3 in H9c2 treated with or without 50 µM Bafilomycin (Baf) and subjected to normoxia or H/R. (J) Pearson correlation of LC3 and PLIN5. (K) MFI quantification of LC3. (L) MFI quantification of PLIN5. (M) SDS‐PAGE and immunoblot analysis of H9c2 lysate treated with or without 10 µM Lalistat 1 (LALi), 100 µM paraoxon (Para), 20 µM atglistatin (ATGL), combination of ATGL and LALi, and combination of ATGL and Para and subjected to normoxia or H/R, using PLIN5, PLIN2, LC3, p62, and GAPDH was used as a loading. (N) Quantification of PLIN5. Scale bar: 10 µm. Results are presented as mean ± SEM (*n* = 3 per group). *p < 0.05, **p < 0.01, ***p < 0.001 versus normoxia control. One‐way ANOVA was run for statistical analysis. Two‐way ANOVA was run when comparing groups on two different categorical variables.
**Figure S3**: H/R altered fatty acid transport, oxidation, and mitochondrial bioenergetics in an autophagy‐dependent manner. (A–E) mRNA expression of CD36, FABP4, CPT1⍺, FATP1, and Rab7 in H9c2 cells subjected to normoxia or H/R. (F) SDS‐PAGE and immunoblot analysis of H9c2 lysate CPT1⍺, GAPDH was used as loading control. (G) Quantification of CPT1⍺. (H) Extracellular free fatty acid measurement in culture medium of H9c2 cells subjected to normoxia or H/R. (I) ATP measurement in culture medium of H9c2 cells subjected to normoxia or H/R. (J) Seahorse mitochondrial stress test and quantification for parameters of mitochondrial function in WT treated with or without chloroquine (CQ), or ATG7 KO cell (AKO) and subjected to normoxia or H/R. Oil: oligomycin; FCCP: carbonyl cyanide‐p‐(trifluoromethoxy) phenylhydrazone; Rot: rotenone; AA: antimycin. (K) Quantification of maximal respiration capacity in Seahorse experiment. Scale bar: 10 µm. Results are presented as mean ± SEM (*n* = 3–12 per group). *p < 0.05, **p < 0.01, ***p < 0.001 versus normoxia control. One‐way ANOVA was run for statistical analysis. Two‐way ANOVA was run when comparing groups on two different categorical variables.
**Figure S4**: H/R disrupted mitochondrial dynamics, mitophagy signaling, and transcriptional regulation of lipid metabolism in H9c2 cells. (A) SDS‐PAGE and immunoblot analysis of H9c2 lysate using p‐Drp1 s616, p‐Drp1 s637, and total Drp1, GAPDH was used as a loading control. (B and C) Quantification of p‐Drp1 s616 and p‐Drp1 s637. (D) SDS‐PAGE and immunoblot analysis of H9c2 lysate using MFF and MFN2, and β‐tubulin was used as loading control. (E and F) Quantification of MFF and MFN2. (G and H) mRNA expression of PPAR⍺, and RXR⍺ in H9c2 cells subjected to normoxia or H/R. (I) SDS‐PAGE and immunoblot analysis of mitochondrial fraction lysate from H9c2 using LC3, p62, PAKIN, and VDAC was used as loading control. (J and K) Quantification of LC3, p62, and PARKIN. (M) Representative image of Mitotrakcer (MT) staining in H9c2 cells treated with or without 10 µM Mdivi‐1 and subjected to normoxia or H/R. (N–P) Quantification of mitochondria density, branch length, and networking. Scale bar: 10 µm. Results are presented as mean ± SEM (*n* = 3) per group. *p < 0.05, **p < 0.01, ***p < 0.001 versus normoxia control. One‐way ANOVA was run for statistical analysis. Two‐way ANOVA was run when comparing groups on two different categorical variables.
**Figure S5**: Rab8a regulated lipid droplet–mitochondria crosstalk, long‐chain fatty acid trafficking, and proteomic remodeling under H/R stress. (A) SDS‐PAGE and immunoblot analysis of Rab8a KO H9c2 cell (RKO) lysate using Rab8a, and β‐tubulin was used as loading control. (B) Quantification of Rab8a. (C) Heat map hierarchical clustering of proteomics analysis of WT and RKO cells. (D) PCA of proteomics analysis of WT and RKO cells (E) Representative image of MT and BODIPY staining in WT and RKO subjected to normoxia or H/R. (F) Pearson correlation of LD and MT. (G) Representative image of LCFA and BODIPY staining in WT and RKO. (H) Pearson correlation of LCFA and LD. (H) Representative image of LCFA and MT staining in WT and RKO. (I) Pearson correlation of LCFA and MT. (J) Representative image of LCFA staining in WT and RKO subjected to normoxia or H/R. (K) Quantification of LCFA MFI. (L) Immunoprecipitation (IP) of Rab8a or IgG from lysates of H9c2 cells or whole cell lysates (WCL) subjected to normoxia or H/R and then blotted for Tom20 and PLIN5. (M) IP of Rab8a from lysates of H9c2 cells or whole cell lysates (WCL) subjected to normoxia or H/R and then blotted for Tom20 and PLIN5. Scale bar: 10 µm. Results are presented as mean ± SEM (*n* = 3 per group). *p < 0.05, **p < 0.01, ***p < 0.001 versus normoxia control. One‐way ANOVA was run for statistical analysis. Two‐way ANOVA was run when comparing groups on two different categorical variables.
**Figure S6**: ALY688 enhanced Rab8a‐associated lipid handling and preserves mitochondrial function during H/R injury. (A) SDS‐PAGE and immunoblot analysis of H9c2 cells lysate using Rab8a, and β‐tubulin was used as loading control. (B) Quantification of Rab8a. (C) IP of Rab8a from lysates of H9c2 cells or whole cell lysates (WCL) subjected to normoxia or H/R and treated with ALY688, and then blotted for Tom20 and PLIN5. (D) Seahorse mitochondrial stress test and quantification for parameters of mitochondrial function in H9c2 treated with or without 100 nM ALY688 and subjected to normoxia or H/R. Oil: oligomycin; FCCP: carbonyl cyanide‐p‐(trifluoromethoxy) phenylhydrazone; Rot: rotenone; AA: antimycin. (E) Quantification of maximal respiration capacity in Seahorse experiment. (F) Representative image of LCFA (long chain fatty acid) and BODIPY staining in H9c2 treated with or without 100 nM ALY688 and subjected to normoxia or H/R. (G) Pearson correlation of FA and BODIPY. Scale bar: 10 µm. Results are presented as mean ± SEM (*n* = 3–4 per group). *p < 0.05, **p < 0.01, ***p < 0.001 versus normoxia control. One‐way ANOVA was run for statistical analysis. Two‐way ANOVA was run when comparing groups on two different categorical variables.

## Data Availability

The data used to support the findings of this study are available from the corresponding author or first author upon reasonable request.
