# Supplementary material for: Loss of Rab8a‐Dependent Tethering of Lipid Droplets to Mitochondria Contributes to Hypoxia–Reoxygenation Injury
Source: MedComm (2020). 2026 Aug 6;7(8):e70845. doi: 10.1002/mco2.70845 (PMC13448150; doi:10.1002/mco2.70845)
Supplement: Supplementary file 1 [file MCO2-7-e70845-s001.pdf]

Loss of Rab8a-Dependent Tethering of Lipid Droplets to Mitochondria Contributes to Hypoxia-Reoxygenation Injury

Jialing Tang[1], Nathan Joyce[2], Thomas Laval[2], Eddie Tam[1], Hye Kyoung Sung[1], Mireille Ouimet[2], Gary Sweeney[1#]

[1]Department of Biology, Faculty of Science, York University, Toronto, Ontario, Canada
[2]Department of Biochemistry, Microbiology and Immunology, Faculty of Medicine, University of Ottawa Heart Institute, Ottawa, Ontario, Canada

# Corresponding author. Department of Biology, Faculty of Science, York University, Toronto, ON, M3J1P3, Canada. Tel 416-736-2100. Email: gsweeney@yorku.ca

Figure S1

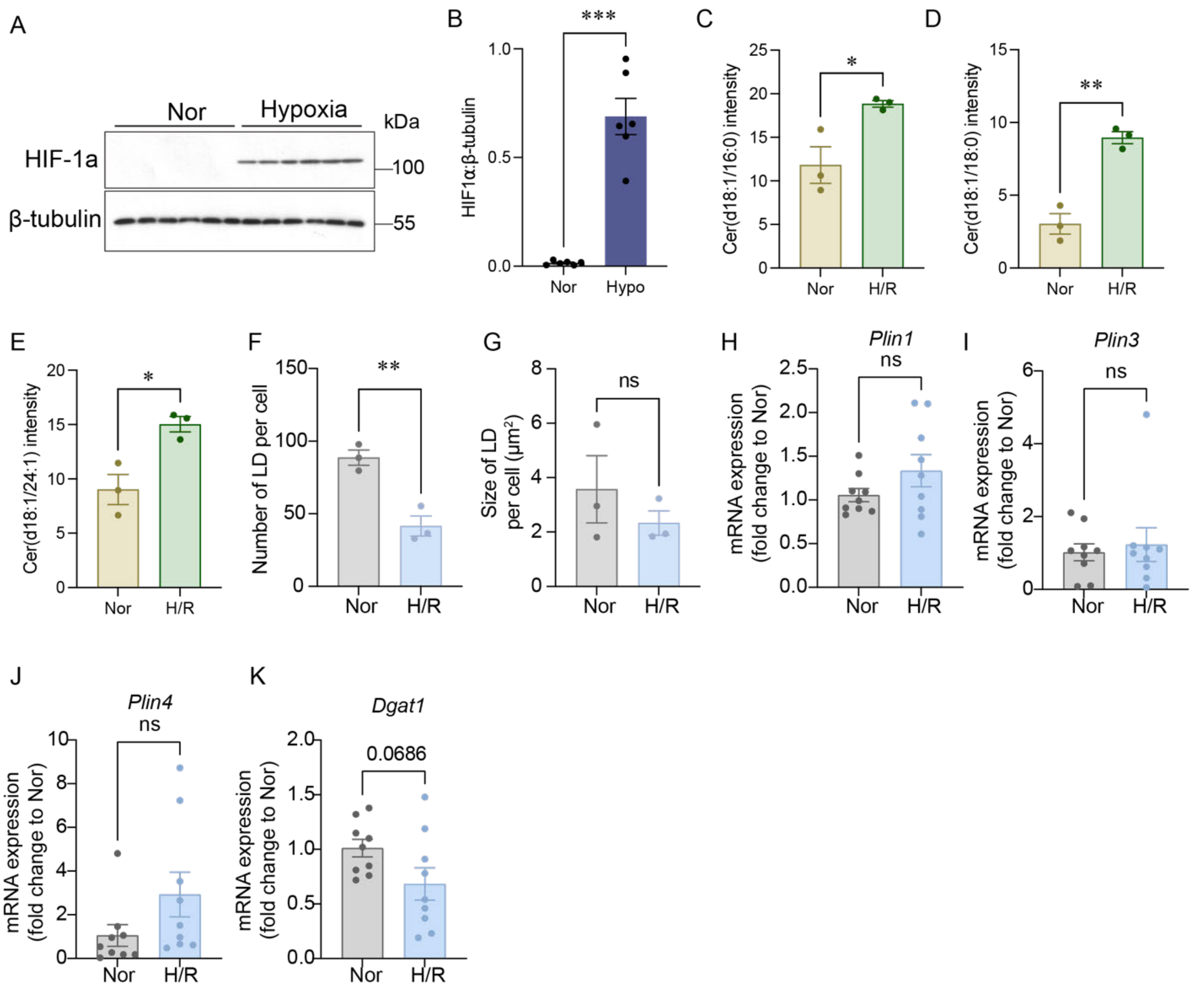

Figure S1 H/R altered ceramide species, and remodels lipid droplet dynamics and lipid metabolism gene expression in H9c2 cells

(A) SDS-PAGE and immunoblot analysis of H9c2 lysate using HIF1α, β-tubulin was used as a loading control. (B) Quantification of HIF1α. (C) Intensity of Cer(d18:1/16:0). (D) Intensity of Cer(d18:1/18:0). (E) Intensity of Cer(d18:1/24:1). (F) Quantification of number of LD of H9c2 cells subjected to normoxia or H/R. (G) Quantification of size of LD of H9c2 cells subjected to normoxia or H/R. (H-K) mRNA expression of PLIN1, PLIN3, PLIN4, DGAT1 in H9c2 cells subjected to normoxia or H/R. Results are presented as mean ± SEM (n=3-9 per group). *P<0.05, **P<0.01, ***P<0.001 versus normoxia control. One-way ANOVA was run for statistical analysis.

Figure S2

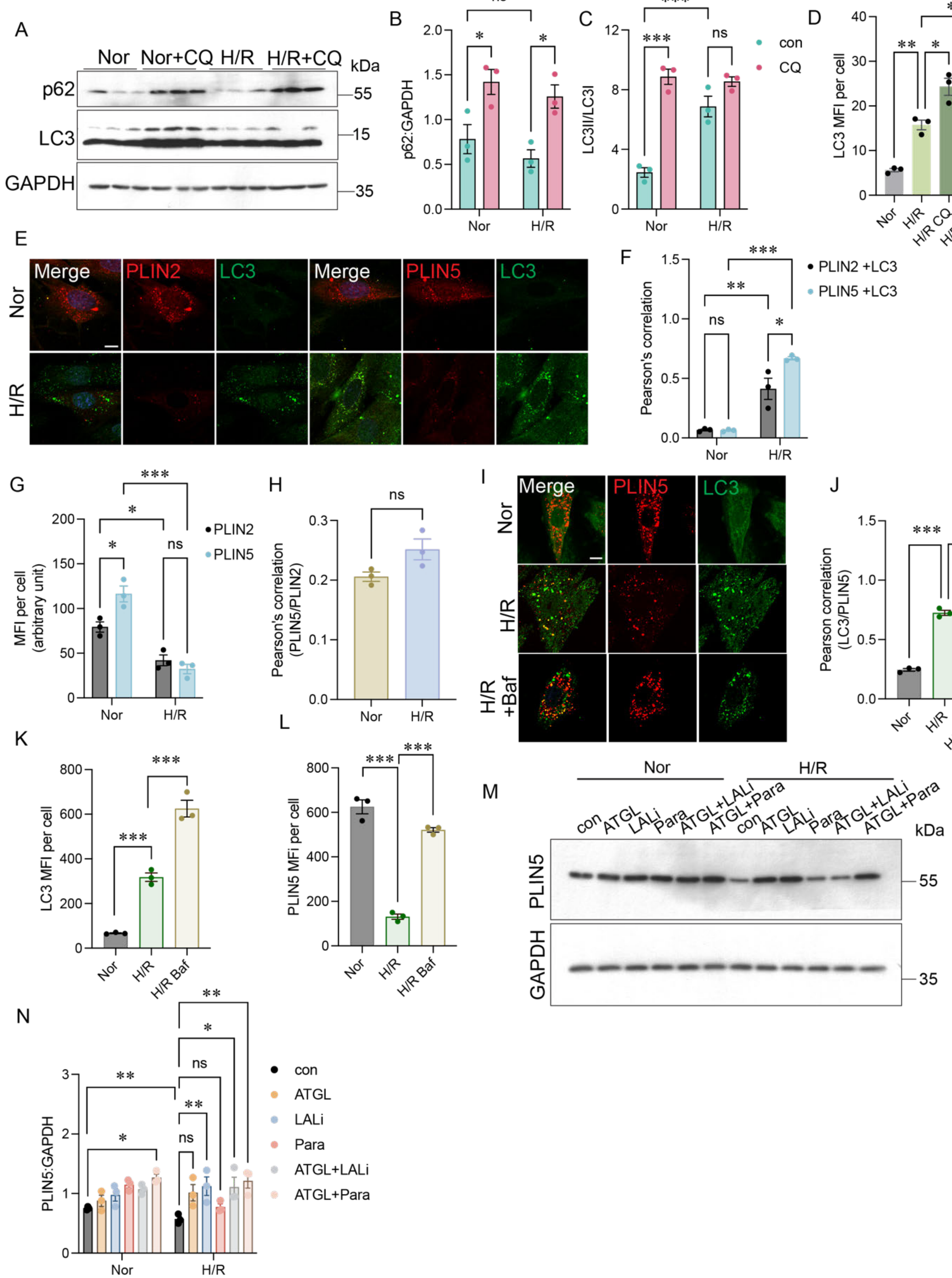
A
Nor Nor+CQ H/R H/R+CQ kDa
p62
LC3
GAPDH
55
15
35
B
p62:GAPDH
Nor H/R
C
LC3II/LC3I
con
CQ
D
LC3 MFI per cell
Nor H/R H/R CQ H/R Baf
E
Merge PLIN2 LC3 Merge PLIN5 LC3
Nor
H/R
F
Pearson's correlation
PLIN2 +LC3
PLIN5 +LC3
G
MFI per cell (arbitrary unit)
PLIN2
PLIN5
H
Pearson's correlation (PLIN5/PLIN2)
I
Merge PLIN5 LC3
Nor
H/R
H/R +Baf
J
Pearson correlation (LC3/PLIN5)
K
LC3 MFI per cell
L
PLIN5 MFi per cell
M
Nor H/R
con ATGL LALi Para ATGL+LALi ATGL+Para con ATGL LALi Para ATGL+LALi ATGL+Para
kDa
PLIN5
GAPDH
55
35
N
PLIN5:GAPDH
con
ATGL
LALi
Para
ATGL+LALi
ATGL+Para

Figure S2 H/R promoted lipid droplet-associated autophagy and PLIN–LC3 interactions in H9c2 cells, modulated by lysosomal and lipolytic pathways

(A) SDS-PAGE and immunoblot analysis of H9c2 lysate using p62 and LC3, GAPDH was used as a loading control. (B-C) Quantification of p62 and LC3. (D) MFI quantification of LC3. (E) Representative image of immunofluorescence staining of LC3 and PLIN2 and PLIN5 in H9c2 subjected to normoxia or H/R. (F) Pearson correlation of PLIN2 and LC3, PLIN5 and LC3. (G) MFI quantification of PLIN2 and PLIN5. (H) Pearson correlation of PLIN2 and PLIN5. (I) Representative image of immunofluorescence staining of PLIN5 and LC3 in H9c2 treated with or without50 µM Bafilomycin and subjected to normoxia or H/R. (J) Pearson correlation of LC3 and PLIN5. (K) MFI quantification of LC3. (L) MFI quantification of PLIN5. (M) SDS-PAGE and immunoblot analysis of H9c2 lysate treated with or without 10 µM Lalistat 1, 100 µM Paraoxon, 20 µM Atglistatin, combination of Atglistatin and Lalistat 1, and combination of Atglistatin and Paraoxon and subjected to normoxia or H/R, using PLIN5, PLIN2, LC3, p62, and GAPDH was used as a loading. (N) Quantification of PLIN5. Scale bar: 10 µm. Results are presented as mean ± SEM (n=3 per group). **P*<0.05, ***P*<0.01, ****P*<0.001 versus normoxia control. One-way ANOVA was run for statistical analysis. Two-way ANOVA was run when comparing groups on two different categorical variables.

Figure S3

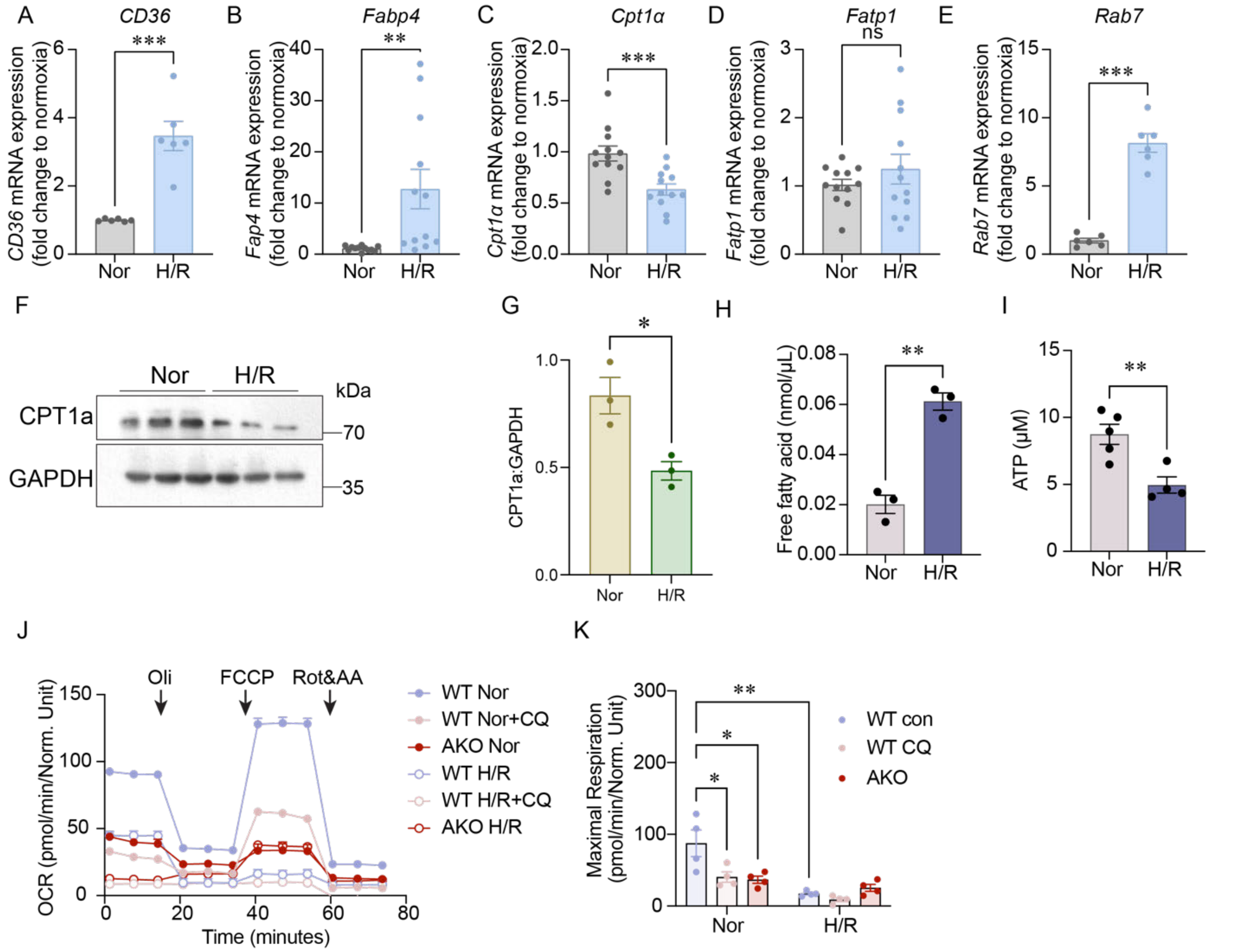

Figure S3 H/R altered fatty acid transport, oxidation, and mitochondrial bioenergetics in an autophagy-dependent manner.

(A-E) mRNA expression of CD36, FABP4, CPT1α, FATP1, and Rab7 in H9c2 cells subjected to normoxia or H/R. (F) SDS-PAGE and immunoblot analysis of H9c2 lysate CPT1α, GAPDH was used as loading control. (G) Quantification of CPT1α. (H) Extracellular free fatty acid measurement in culture medium of H9c2 cells subjected to normoxia or H/R. (I) ATP measurement in culture medium of H9c2 cells subjected to normoxia or H/R. (J) Seahorse mitochondrial stress test and quantification for parameters of mitochondrial function in WT treated with or without chloroquine, or ATG7 KO and subjected to normoxia or H/R. (K) Quantification of maximal respiration capacity in Seahorse experiment. Scale bar: 10 μm. Results are presented as mean ± SEM (n=3-12 per group). *P<0.05, **P<0.01, ***P<0.001 versus normoxia control. One-way

ANOVA was run for statistical analysis. Two-way ANOVA was run when comparing groups on two different categorical variables.

Figure S4

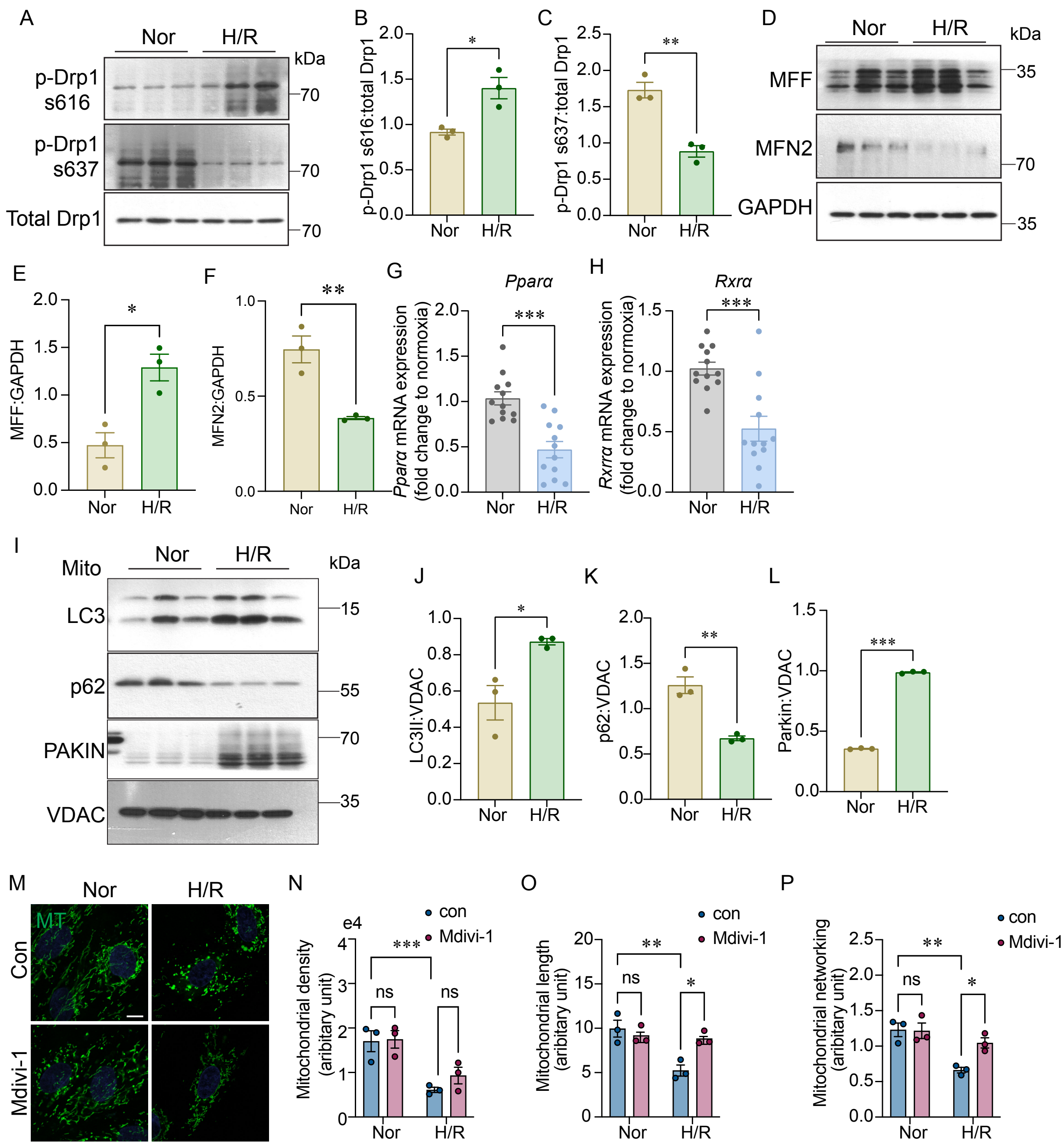

Figure S4 H/R disrupted mitochondrial dynamics, mitophagy signaling, and transcriptional regulation of lipid metabolism in H9c2 cells

(A) SDS-PAGE and immunoblot analysis of H9c2 lysate using p-Drp1 s616, p-Drp1 s637 and total Drp1, GAPDH was used as a loading control. (B-C) Quantification of p-Drp1 s616, and p-Drp1

s637. (D) SDS-PAGE and immunoblot analysis of H9c2 lysate using MFF and MFN2, and β-tubulin was used as loading control. (E-F) Quantification of MFF and MFN2. (G-H) mRNA expression of PPARα, and RXRα in H9c2 cells subjected to normoxia or H/R. (I) SDS-PAGE and immunoblot analysis of mitochondrial fraction lysate from H9c2 using LC3, p62, PAKIN, and VDAC was used as loading control. (J-K) Quantification of LC3, p62 and PARKIN. (M) Representative image of Mitotrakcer (MT) staining in H9c2 cells treated with or without 10 µM Mdivi-1 and subjected to normoxia or H/R. (N-P) Quantification of mitochondria density, branch length, and networking. Scale bar: 10 µm. Results are presented as mean ± SEM (n=3) per group. *P<0.05, **P<0.01, ***P<0.001 versus normoxia control. One-way ANOVA was run for statistical analysis. Two-way ANOVA was run when comparing groups on two different categorical variables.

Figure S5

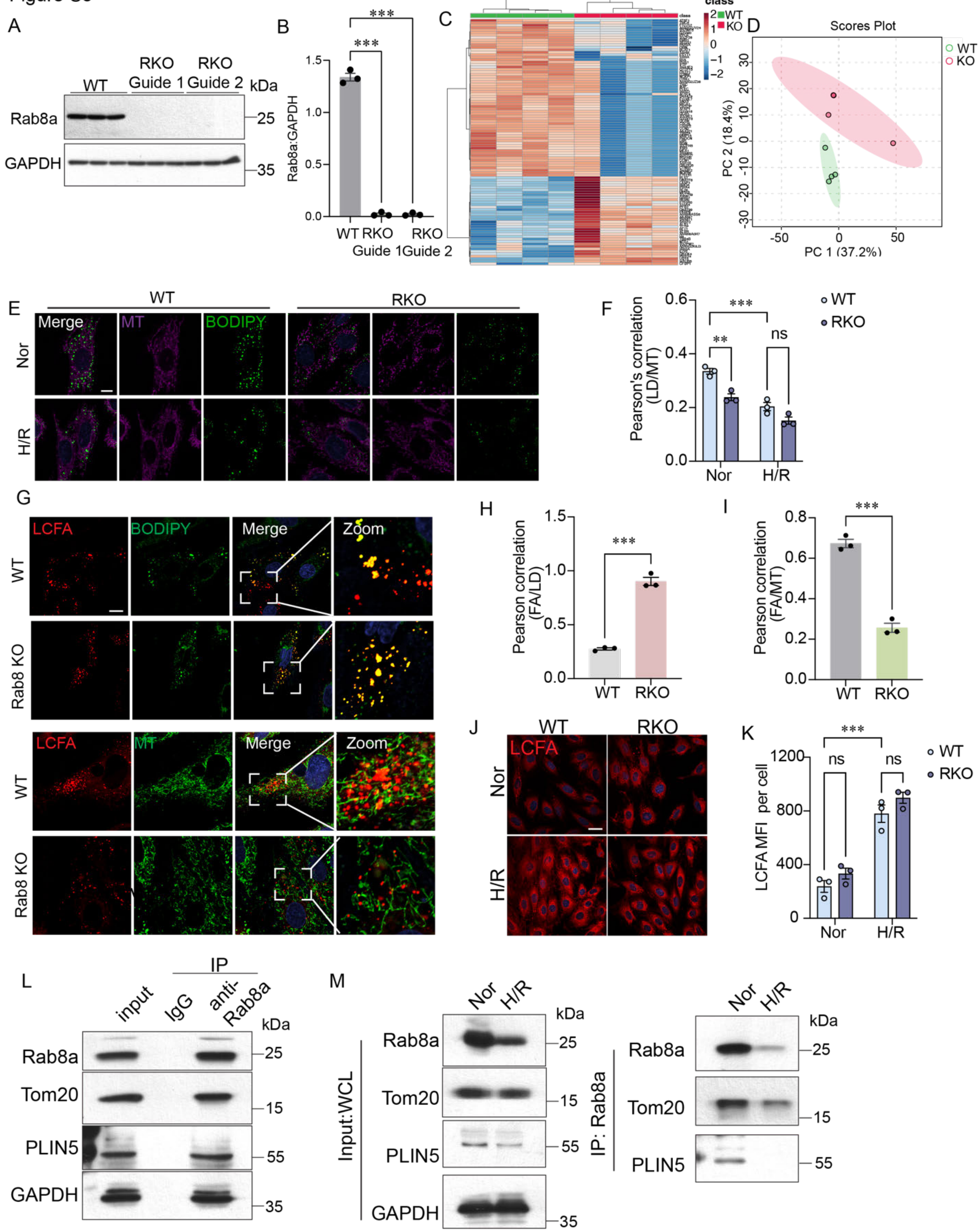
A
WT
RKO Guide 1
RKO Guide 2
kDa
Rab8a
GAPDH
25
35
B
Rab8a:GAPDH
WT
RKO Guide 1
RKO Guide 2
C
class
WT
KO
D
Scores Plot
PC 1 (37.2%)
PC 2 (18.4%)
E
WT
RKO
Merge
MT
BODIPY
Nor
H/R
F
Pearson's correlation (LD/MT)
ns
G
LCFA
BODIPY
Merge
Zoom
WT
Rab8 KO
MT
H
Pearson correlation (FA/LD)
I
Pearson correlation (FA/MT)
J
K
LCFA MFI per cell
L
IP
input
IgG
anti-Rab8a
Tom20
PLIN5
M
Input:WCL
IP: Rab8a

Figure S5 Rab8a regulated lipid droplet-mitochondria crosstalk, long-chain fatty acid trafficking, and proteomic remodeling under H/R stress

(A) SDS-PAGE and immunoblot analysis of RKO lysate using Rab8a, and β-tubulin was used as loading control. (B) Quantification of Rab8a. (C) Heat map hierarchical clustering of proteomics analysis of WT and RKO cells. (D) PCA of proteomics analysis of WT and RKO cells (E) Representative image of MT and BODIPY staining in WT and RKO subjected to normoxia or H/R. (F) Pearson correlation of LD and MT. (G) Representative image of LCFA and BODIPY staining in WT and RKO. (H) Pearson correlation of LCFA and LD. (H) Representative image of LCFA and MT staining in WT and RKO. (I) Pearson correlation of LCFA and MT. (J) Representative image of LCFA staining in WT and RKO subjected to normoxia or H/R. (K) Quantification of LCFA MFI. (L) Immunoprecipitation (IP) of Rab8a or IgG from lysates of H9c2 cells or whole cell lysates (WCL) subjected to normoxia or H/R and then blotted for Tom20 and PLIN5. (M) IP of Rab8a from lysates of H9c2 cells or whole cell lysates (WCL) subjected to normoxia or H/R and then blotted for Tom20 and PLIN5. Scale bar: 10 µm. Results are presented as mean ± SEM (n=3 per group). **P*<0.05, ***P*<0.01, ****P*<0.001 versus normoxia control. One-way ANOVA was run for statistical analysis. Two-way ANOVA was run when comparing groups on two different categorical variables.

Fgure S6

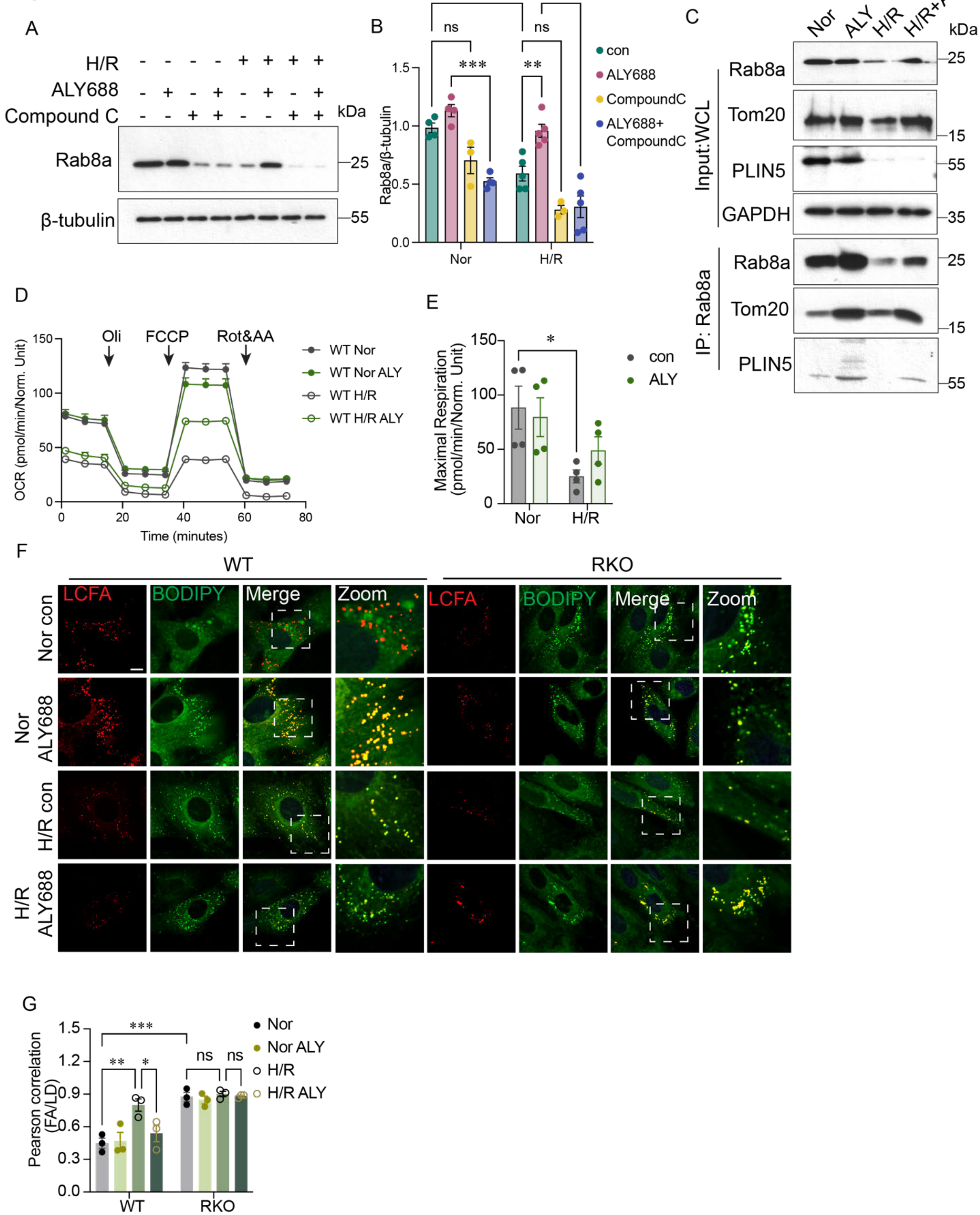
A
H/R
ALY688
Compound C
kDa
Rab8a
25
β-tubulin
55
B
Rab8a/β-tubulin
Nor
H/R
con
ALY688
CompoundC
ALY688+ CompoundC
ns
**
***
C
Nor
ALY
H/R
H/R+ALY
kDa
Input:WCL
IP: Rab8a
Rab8a
Tom20
PLIN5
GAPDH
25
15
55
35
D
Oli
FCCP
Rot&AA
OCR (pmol/min/Norm. Unit)
Time (minutes)
WT Nor
WT Nor ALY
WT H/R
WT H/R ALY
E
Maximal Respiration (pmol/min/Norm. Unit)
con
ALY
Nor
H/R
F
WT
RKO
LCFA
BODIPY
Merge
Zoom
Nor con
Nor ALY688
H/R con
H/R ALY688
G
Pearson correlation (FA/LD)
Nor
Nor ALY
H/R
H/R ALY
WT
RKO

Figure S6 ALY688 enhanced Rab8a-associated lipid handling and preserves mitochondrial function during H/R injury

(A) SDS-PAGE and immunoblot analysis of H9c2 cells lysate using Rab8a, and β-tubulin was used as loading control. (B) Quantification of Rab8a. (C) IP of Rab8a from lysates of H9c2 cells or whole cell lysates (WCL) subjected to normoxia or H/R and treated with ALY688, and then blotted for Tom20 and PLIN5. (D) Seahorse mitochondrial stress test and quantification for parameters of mitochondrial function in H9c2 treated with or without 100 nM ALY688 and subjected to normoxia or H/R. (E) Quantification of maximal respiration capacity in Seahorse experiment. (F) Representative image of long LCFA and BODIPY staining in H9c2 treated with or without 100 nM ALY688 and subjected to normoxia or H/R. (G) Pearson correlation of FA and BODIPY. Scale bar: 10 μm. Results are presented as mean ± SEM (n=3-4 per group). *P<0.05, **P<0.01, ***P<0.001 versus normoxia control. One-way ANOVA was run for statistical analysis. Two-way ANOVA was run when comparing groups on two different categorical variables.

Table S1: Sequences of primers

Target	Species	Forward 5'-3'	Reverse 5'-3'
Dgat1	Rat	GAACTAGCTAGCCGTCCGTC	AAAGGATCTGCCCTGTCACG
Dgat2	Rat	ACCACGCACCAATTCAGGAT	TCCTTTTCACCAGGTGCCAT
Plin1	Rat	GTGCCATCGTCTGCAAGATTC	GCATCACCACACACCAATTCAG
Plin2	Rat	GCGCTACTTCCGAGACTACTT	GGGCCTTATGCCAGGAAACT
Plin3	Rat	GAGTCACAACCCCACGATGT	CGAGAGAGGAAAGAGTCGAC
Plin4	Rat	CAGTACTTGCCGCTCACTCA	TCAGATGGACAGTGGAGTGG
Pline5	Rat	CTCAGTGTCTGGTGCAAAGG	CTAGTTCTGTGTCTGTCAGG
Atgl	Rat	GGCTACCGAGATGGACTTCG	AGACTCCGTCTCCCATCCTC
Hsl	Rat	ATGCTGTGTGAGAATGCCGA	GGCCTGACAGAGCAAAGAGT
Mgl	Rat	ACTAATGGAAACAGGGCCCAA	TGTCCTGACTCGGGGATGAT
Lal	Rat	GGTACACTGGACCCAGGTTG	GATGTCACTGGTGTCTGCCA
Cpt1α	Rat	CCAGGCTACAGTGGGACATT	GAAGAGCCGAGTCATGGAAG
Cd36	Rat	ACAGATGCAGCCTCCTTTCC	ACACAGCATAGATGGACCTGC
Fatp1	Rat	TCTACCCTTCTGGCTCCCAT	TCTACCAGGTGTCAAGGCTCA
Fabp4	Rat	AGA AGT GGG AGT TGG CTT CG	ACT CTC TGA CCG GAT GAC GA
Rxrα	Rat	CAGGGGATGCACAACGCTGGC	CCGACTGTCCGCTTAGAG
Pparα	Rat	TAGCAACAATCCGCCTTTTGT	GCGATCAGCATCCCGTCTT
